# Methamphetamine-induced region-specific transcriptomic and epigenetic changes in the brain of male rats

**DOI:** 10.1038/s42003-023-05355-3

**Published:** 2023-09-27

**Authors:** Benpeng Miao, Xiaoyun Xing, Viktoriia Bazylianska, Pamela Madden, Anna Moszczynska, Bo Zhang

**Affiliations:** 1 Department of Developmental Biology, Center of Regenerative Medicine, Washington University School of Medicine, St. Louis, MO 63110 USA; 2 Department of Genetics, Center for Genomic Sciences and Systems Biology, Washington University School of Medicine, St. Louis, MO 63110 USA; 3 Department of Pharmaceutical Sciences, Wayne State University, Detroit, MI 48201 USA; 4 Department of Psychiatry, Washington University School of Medicine, St. Louis, MO 63110 USA

**Keywords:** Computational biology and bioinformatics, Epigenetics

## Abstract

Psychostimulant methamphetamine (METH) is neurotoxic to the brain and, therefore, its misuse leads to neurological and psychiatric disorders. The gene regulatory network (GRN) response to neurotoxic METH binge remains unclear in most brain regions. Here we examined the effects of binge METH on the GRN in the nucleus accumbens, dentate gyrus, Ammon’s horn, and subventricular zone in male rats. At 24 h after METH, ~16% of genes displayed altered expression and over a quarter of previously open chromatin regions - parts of the genome where genes are typically active - showed shifts in their accessibility. Intriguingly, most changes were unique to each area studied, and independent regulation between transcriptome and chromatin accessibility was observed. Unexpectedly, METH differentially impacted gene activity and chromatin accessibility within the dentate gyrus and Ammon’s horn. Around 70% of the affected chromatin-accessible regions in the rat brain have conserved DNA sequences in the human genome. These regions frequently act as enhancers, ramping up the activity of nearby genes, and contain mutations linked to various neurological conditions. By sketching out the gene regulatory networks associated with binge METH in specific brain regions, our study offers fresh insights into how METH can trigger profound, region-specific molecular shifts.

## Abstract (web summary)

A multi-omic analysis unveils the transcriptomic and epigenomic impact of acute, peripheral methamphetamine exposure on reward-related brain regions in adult, male rats.

## Introduction

Methamphetamine (METH) is a powerful and widely used psychostimulant that has deleterious effects on the central nervous system and results in addiction, which is a major public concern globally^1–4^. In the United States, close to 2,000,000 people who misuse METH, and deaths from METH misuse are rapidly rising^5^. Between 2015 and 2019, METH misuse increased by 43%, while the number of people suffering from METH misuse disorder increased by 62%. The number of deaths from METH misuse increased tenfold between 2009 and 2019^6^. METH misuse, particularly at high doses, is associated with neurologic and psychiatric disorders, as it causes severe cognitive impairment and neurobehavioral abnormalities^7,8^. Recent studies have shown the acute and long-term effects of METH on cognitive functions such as attention, working memory, and learning, and METH overdose is often fatal^9–14^. Meanwhile, the high relapse frequency of METH misuse is a crucial challenge for treating METH use disorder^15–17^. There is no FDA-approved medication for METH misuse disorder, highlighting the importance of better understanding the molecular mechanism of the brain’s reaction to METH exposure and particularly to METH high-dose exposure, which causes neurotoxicity in the reward circuitry.

The reward circuitry plays a central role in different substance use disorders. The circuitry encompasses multiple brain subregions, including the ventral tegmental area, nucleus accumbens (NAc), dorsal striatum, amygdala, hippocampus, and regions of the prefrontal cortex^18–21^. In these different brain regions, distinct significant transcriptional and epigenetic changes can be caused by different addictive substances^18,22–28^, creating complicated crosstalk between the epigenetic landscape and transcriptome in the brain^29,30^. For example, acute or chronic exposure of the brain to psychostimulants, including METH, opiates, and alcohol can upregulate histone acetyltransferases while suppressing histone deacetylases, resulting in increased acetylation levels of histones H3 and H4 in the NAc and subsequent upregulation of their target genes^31–38^. METH exposure increased the acetylation levels of H4K5 and H4K8 in gene promoter regions in the rat striatum^39^ and NAc^40^. Addictive drugs can also influence the expression of DNA methyltransferases and further induce changes in DNA methylation, which plays essential roles in cognitive learning and memory^41,42^. Acute and chronic METH injections can increase DNMT1 expression in the rat NAc and dorsal striatum^43^, and METH self-administration can increase the DNA methylation level of several potassium channel genes in the rat brain^44^.

Recently, assay for transposase-accessible chromatin with high-throughput sequencing (ATAC-seq), a method for mapping genome-wide chromatin accessibility, has been widely used in addiction research to explore the open chromatin regions (OCRs) associated with exposure to addictive substances^45–48^, but not including METH. The changes in chromatin accessibility in OCRs interact directly with histone modifications and DNA methylation and are usually associated with the binding of different transcription factors, which are the regulatory hubs in response to substance exposure. Many transcription factors, such as ΔFOSB, early growth response factors, and multiple myocyte-specific enhancer factor 2, were found to respond to addictive substance stimuli and to regulate their downstream target genes^18,22,49–53^. However, most of these studies focused on a single brain region, such single-tissue and single-omics strategies generate many difficulties in directly comparing the molecular changes responding to the misuse of substances across multiple brain regions, and result in the difficulty for multi-omics data integration across different studies. There is still a vast gap in understanding the simultaneous molecular changes and neurotoxicity in different brain regions upon substance stimuli.

In this study, we analyzed the molecular changes of the transcriptome and chromatin accessibility after exposure to neurotoxic METH regiment (binge METH) in four brain regions of male rats, including the NAc), dentate gyrus (DG), Ammon’s horn (*Cornu Ammonis*, CA), and subventricular zone (SVZ). The SVZ and the subgranular zone of the DG are the only active neurogenic areas in the adult brain. Exposure to METH affects adult neurogenesis in the SVZ and subgranular zone^54,55^, while modulation of subgranular zone and SVZ neurogenesis impacts hippocampal-based cognitive function^56^. The CA and DG work together to process and integrate spatial and contextual information, ultimately facilitating the encoding, storage, and retrieval of long-term memories^57,58^. Dysfunctions in these regions are associated with various neurological disorders, such as Alzheimer’s disease, epilepsy, and schizophrenia. How the different brain regions simultaneously respond to the neurotoxic acute exposure of high doses METH at both transcriptomic and epigenetic levels, is still largely unknown.

In our study, we determined that METH exposure induced a total of 2254 differentially expressed genes (DEGs) and 25,598 differentially accessible regions (DARs) in the four rat brain regions. These four rat brain regions generally displayed a strong region-specific response to METH exposure at both the transcriptomic and epigenetic levels. Few of those DEGs and DARs were simultaneously affected by METH exposure in all four regions. We observed an interesting opposite regulation pattern between the CA and DG regions: 119 genes and 764 OCRs were significantly oppositely regulated by METH exposure.

Furthermore, METH exposure significantly affected the expression of 146 transcription factors (TFs) and 31 epigenetic modification factors (Epi-Modifier), which could initiate epigenetic remodeling of chromatin and further regulate target gene expression. For accessible DARs identified in four regions, ~70% of them were conserved orthologous in rat, mouse, and human genomes. The genes around these conserved DARs were highly enriched in the neurological processes. Some orthologous DARs in mouse and human genome regions could intersect with validated enhancers, and GWAS SNPs related to orthologous DARs in mouse and human genome regions could cross with validated enhancers and GWAS SNPs related to neuron biology. Meanwhile, TFs binding motifs enriched in DARs composed distinct gene regulation networks in different brain regions in response to binge METH. Taken together, our study provides a comprehensive multi-omics investigation into the neurotoxic reactions of different brain regions exposed to METH stimulus.

## Results

### Transcriptome and chromatin accessibility signatures of 4 brain regions in normal and METH-exposed rats

**Fig. 1:**
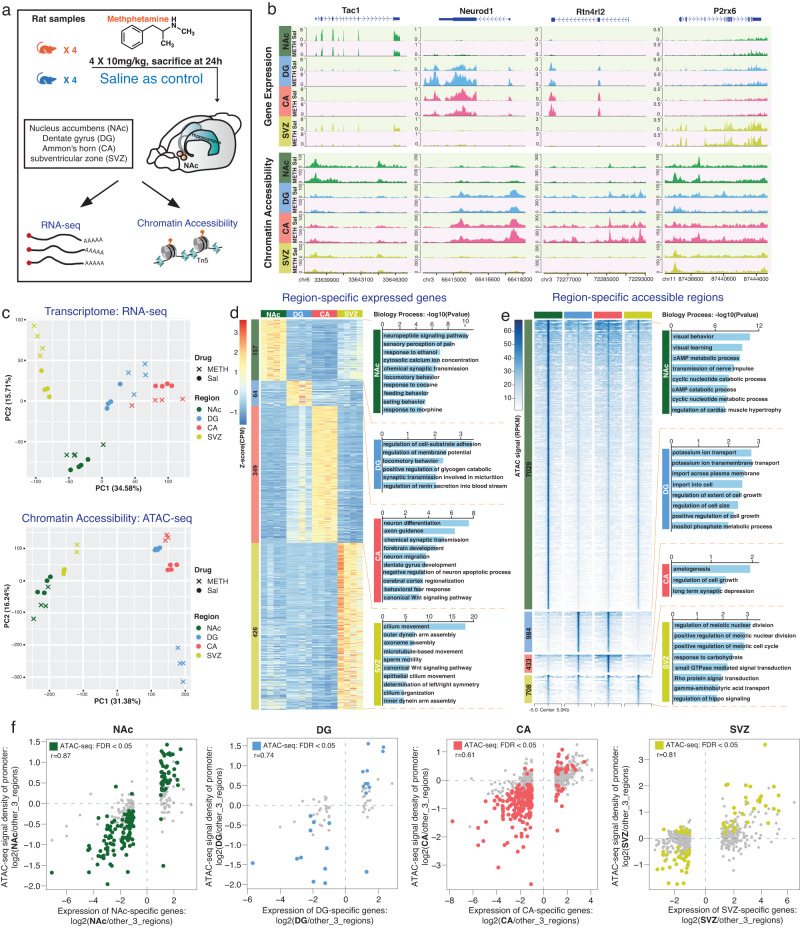
Gene expression and epigenetic signals of 4 brain regions from control and binge METH exposed rat samples. **a** Schematic outline of the experimental design. **b** Examples of gene expression and open chromatin signals in METH exposed and Saline samples of 4 rat brain regions. The *Tac1* gene was more highly expressed and became more open only in the NAc region. The *Neurod1* and *Rtn4rl2* genes showed high expression and chromatin accessibility in both the DG and CA. The *P2rx6* gene showed higher expression only in the SVZ. METH: binge METH exposed samples, red background; Sal: saline samples, green background. **c** The principal analysis of METH exposed and Sal samples of 4 brain regions for RNA-seq and ATAC-seq data. Cross: METH exposed samples; dot: saline samples. The region-specific signatures of 4 brain regions in saline controls at both the transcriptomic (**d**) and epigenetic (**e**) levels. **d** Left part shows the number and expression Z score of region-specific genes in 4 brain regions; **e** Left part shows the number and open chromatin signals of region-specific accessible regions in 4 brain regions. The right parts of (**d** and **e**) show the enriched biological process terms. **f** The relationship between the expression of region-specific genes and ATAC-seq signal on their promotor regions (*n* = 16 saline control samples of ATAC-seq data).

To explore the molecular changes in the brain in response to acute binge METH, we exposed young male Sprague–Dawley rats to high-dose METH injections (4 × 10 mg/kg, every 2 h). Meanwhile, a control group of male rats received saline injections at the same time intervals. The NAc, DG, CA, and SVZ were collected 24 h after the last dose of METH or saline injection. The transcriptome and epigenome of these rat brains were assessed using RNA-seq and ATAC-seq assays (Fig. 1a and Supplementary Data 1). As expected, we observed strong region-specific transcriptome and epigenome profiles in each of the four brain regions (Fig. 1b). Principal component analysis clearly separated these four different rat brain regions in both RNA-seq and ATAC-seq assays (Fig. 1c and Supplementary Fig. 1a, b), emphasizing the existence of strong brain-regional specificity at both the transcriptomic and epigenetic levels. The DG and CA, two connected regions in the hippocampal formation, were closer to each other in the principal component analysis, as expected (Fig. 1c), but showed the opposite response to binge METH, especially at the genome-wide chromatin level (Fig. 1c), suggesting strong epigenetic remodeling happened in METH-exposed DG region.

We first investigated the region-specific signatures of the four brain regions in saline controls at both the transcriptomic and epigenetic levels by identifying the region-specific expressed genes and accessible chromatin regions (Supplementary Fig. 1 and Supplementary Data 2). At the gene level, 157, 64, 349, and 426 region-specific genes were identified separately in the NAc, DG, CA, and SVZ regions, respectively (Fig. 1d and Supplementary Fig. 1c). These genes were significantly enriched in distinct neurological functions and processes in a region-specific fashion (Fig. 1d and Supplementary Data 3). In the NAc region, 10 of 157 genes were directly associated with the drug responses to cocaine and morphine. CA region-specific genes were highly enriched in neuron differentiation and axon guidance. We identified 426 genes explicitly expressed in the SVZ region, and these genes were enriched in movement- and assembly-associated biological functions, suggesting their involvement in neuron differentiation and migration activities in the SVZ region (Fig. 1d). At the epigenetic level, we identified 7029 NAc-specific open chromatin regions (OCRs, Supplementary Fig. 1d). The genes around these NAc-specific OCRs were enriched in behavior, learning, and cAMP metabolic processes (Fig. 1e and Supplementary Data 3). However, only a few hundred region-specific OCRs were identified separately in the other three brain areas (Fig. 1e and Supplementary Fig. 1d). Such results suggested that the NAc brain area is unique at the epigenetic regulatory level. Most of these region-specific OCRs identified in 4 brain regions were far away from gene promoters and located in intronic and intergenic regions (Supplementary Fig. 1e, f). About 30% of mouse orthologs of rat region-specific OCRs played roles as distal enhancers (Supplementary Fig. 1g), furthermore, we found 6–40% of regions-specific DEGs also harbored the regions-specific OCRs (Supplementary Fig. 1h), such results emphasized the importance of enhancer elements in cell fate determination as previously reported^59–61^. We further explored the chromatin accessibility of region-specific genes (Fig. 1f). We noticed the positive correlation between the expression of region-specific genes and ATAC-seq signal on their promoters. The promoter chromatin accessibility of many region-specific genes statistically differed on a relatively small scale for each brain region, when compared to the other three regions (Fig. 1f).

### METH exposure induced region-specific transcriptomic changes in 4 rat brain regions

**Fig. 2:**
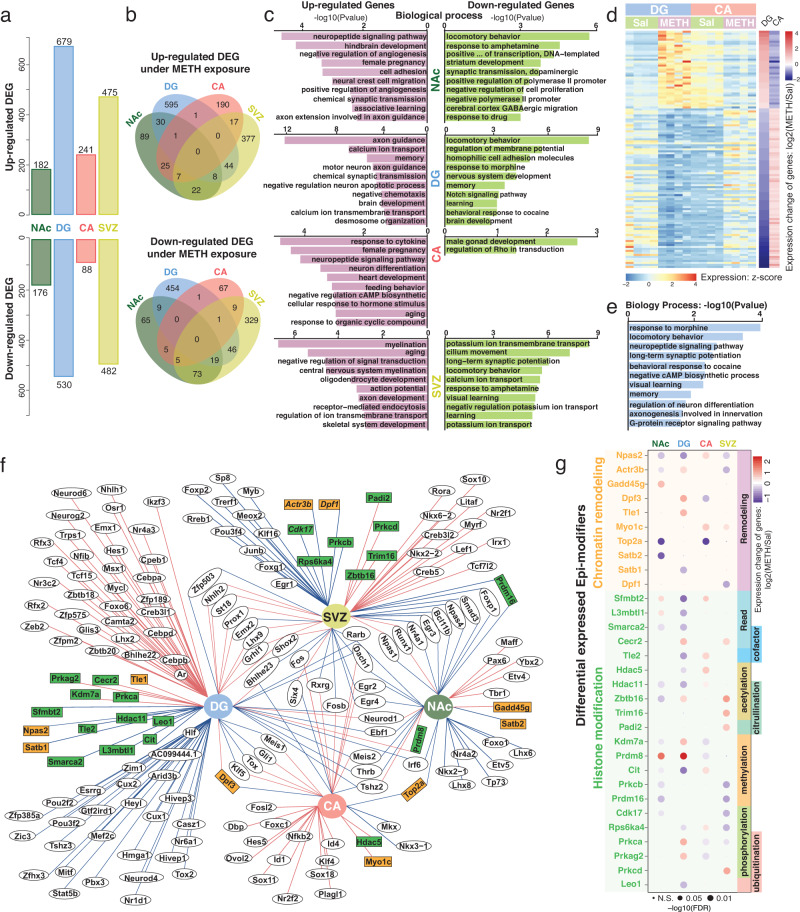
Differentially expressed genes in 4 rat brain regions in response to binge METH stimulus. **a** Number of up- and down-regulated differentially expressed genes (DEGs) induced by METH exposure in 4 brain regions. The largest number of DEGs was identified in the DG region. **b** The number of shared up- and downregulated DEGs across 4 brain regions. Only a few DEGs were simultaneously affected by METH exposure in multiple regions. **c** Gene Ontology (biological process) enrichment analysis of up- and downregulated DEGs in 4 brain regions. **d** The DEGs show opposing regulation patterns between the DG and CA. A total of 39 upregulated DEGs in the DG were identified as downregulated DEGs in the CA, and 80 downregulated DEGs in the DG were identified as upregulated DEGs in the CA. **e** GO enrichment analysis of DEGs showing opposing regulation patterns between the DG and CA. These DEGs were highly enriched in neurological process terms. **f** METH stimulus-induced significant expression changes in 146 transcription factors (TFs) and 31 epigenetic factors (Epi-Modifiers) in 4 brain regions. The red line shows upregulated DEGs, and the blue line shows downregulated DEGs. Genes shown in circles are TFs, and genes shown in squares are Epi-Modifiers. The orange and green background colors match the chromatin remodeling and histone modification functions, respectively, in (**g**). **g** Differentially expressed Epi-Modifiers with chromatin remodeling and histone modification functions in 4 brain regions (*n* = 4 control and 4 METH exposure samples for each brain region).

METH exposure can induce significant global molecular changes in all four brain regions (Fig. 1c). To better characterize the region-specific transcriptomic response to METH stimulus, we identified the differentially expressed genes in each region separately and further checked the chromatin accessibility on the promoter of these METH exposure-induced DEGs (Fig. 2a, Table 1, Supplementary Data 4, and Supplementary Fig. 2a). We identified 1209 METH exposure-induced DEGs in the DG region, the highest number among all four areas (Fig. 2a and Table 1). In the SVZ, 957 genes were significantly changed, suggesting that the SVZ is another vital target brain region for METH stimuli (Fig. 2a and Table 1). In the NAc and CA, only 358 and 329 genes, respectively, exhibited significant changes in expression (Fig. 2a and Table 1). We further cross-referenced the METH exposure-responsive DEGs in all four brain regions and found that most of the DEGs were regionally regulated; only a few genes responded to METH binge in multiple areas, including *Gfap*, *Nrn1*, and *Drd1* (Fig. 2b and Supplementary Fig. 2b). Such results suggested that the significant transcriptomic response to METH stimulus has high regional specificity and different sensitivity to stimulation in distinct brain regions. GO enrichment analysis of the region-specific DEGs revealed their enrichment in certain biological processes, e.g., specifically downregulated DEGs in the NAc, DG, and SVZ were enhanced in locomotory behavior and response to drugs, including amphetamine, morphine, and cocaine (Fig. 2c). METH exposure-induced upregulated DEGs in the NAc, DG, and CA were enriched in essential neuronal functions, such as signaling pathways, neuron development, axon guidance, memory, etc. Upregulated DEGs in the SVZ were highly enriched in myelination and oligodendrocyte development, suggesting that glial cells in the SVZ region have a distinct response to the binge METH (Fig. 2c and Supplementary Data 5).

**Table 1:** The number of differentially expressed genes and differentially accessible regions in 4 brain regions of male rats after the acute binge METH exposure.

Brain regions	Differential analysis of rat brain responding to METH exposure			
	Differential expressed genes (DEGs)		Differential accessible regions (DARs)	
	Up-regulated DEGs	Down-regulated DEGs	More-accessible DARs	Less-accessible DARs
NAc	182	176	708	3907
DG	679	530	3399	12265
CA	241	88	1641	347
SVZ	475	482	4693	1297

The DG and CA are spatially connected regions in the hippocampus and together contribute to new memory formation. However, we only found two commonly upregulated and two commonly downregulated DEGs after the binge METH in the DG and CA (Fig. 2b). Surprisingly, we found that 39 upregulated DEGs in the DG were identified as downregulated DEGs in the CA, and 80 downregulated DEGs in the DG were identified as upregulated DEGs in the CA (Fig. 2d and Supplementary Fig. 2c). These types of DEGs were highly enriched in neurological processes, including response to morphine and cocaine, memory, visual learning, and long-term synaptic potentiation (Fig. 2e and Supplementary Fig. 2d). Such opposite gene regulation in the DG and CA indicated a distinct regulation in the trisynaptic circuit in response to the binge METH stimulus.

Transcription factors (TFs) and epigenetic modification factors (Epi-Modifiers) are critical components in the gene regulatory network. We precisely checked the TFs and Epi-Modifiers associated with the binge METH exposure in all four rat brain regions. In total, we found 146 TFs and 31 Epi-Modifiers that significantly changed expression after the METH stimulus (Fig. 2f and Supplementary Data 4). Twenty-seven TFs changed their expression in more than one brain region. Only seven TFs, including *Egr2*, *Egr4*, *Rarb*, *Bhlhe23*, *Meis2*, *Fosb*, and *Tshz2*, were differentially expressed in more than two brain regions, and *Dach1* was the only TFs that responded to the METH stimulus in all four rat brain regions. Among 31 differentially expressed Epi-Modifiers, 10 Epi-Modifiers are considered to play essential roles in chromatin remodeling, and 21 genes are associated with histone modification, including acetylation, methylation, and phosphorylation (Fig. 2g). Three Epi-Modifier genes, including *Prdm8*, *Dpf3*, and *Top2a*, were found to be significantly changed in more than one brain region (Fig. 2g). For example, *Prdm8*, a conserved histone methyltransferase that acts predominantly as a negative regulator of transcription^62^, was commonly upregulated in the NAc and DG, suggesting that epigenetic regulation might be involved in the repression of genes that respond to METH in these two areas (Fig. 2c and Supplementary Fig. 2e). We further checked the expression changes of these Epi-Modifiers across the four brain regions and found that many of these Epi-Modifier genes showed changes in expression. The *Hdac5* gene, which is responsible for the deacetylation of lysine residues on the N-terminal part of the core histones, showed an opposite regulation pattern between the DG and CA (Supplementary Fig. 2f)^18^. The significant differential expression of Epi-Modifiers in response to binge METH could also result in epigenetic changes.

### METH exposure induced region-specific epigenetic changes in the rat brain

**Fig. 3:**
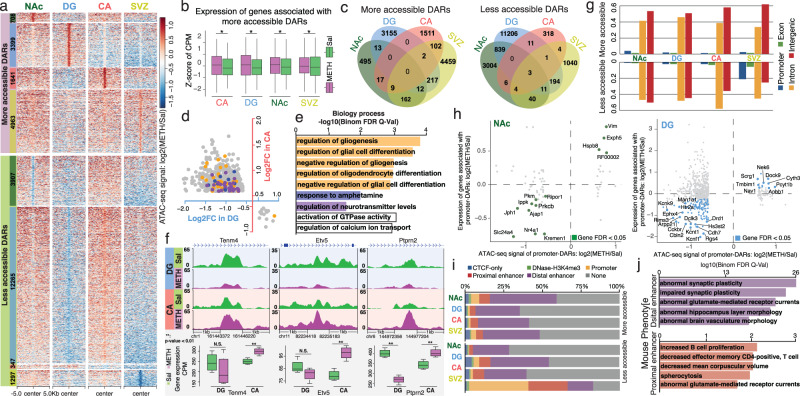
Differentially accessible regions in 4 brain regions induced by binge METH exposure. **a** More and less accessible DARs identified in 4 brain regions in response to binge METH stimulus. Each dot represents a 200 bp window with log2 ratio of ATAC-seq signals by METH-exposed vs. Sal samples. **b** Expression of genes associated with more accessible DARs induced by METH exposure. *: wilcox.test, *p* value < 0.05; *n* = 4 saline and 4 METH exposure samples in different brain regions. **c** The number of shared more and less accessible DARs across 4 rat brain regions. Eighty-nine percent of DARs were identified only in a single brain region. A small number of DARs were identified in multiple regions. **d** The DARs show opposite accessibility changes in the DG and CA in response to binge METH stimulus. A total of 764 more accessible DARs in the CA were identified as less accessible DARs in the DG, and another 16 less accessible DARs in the CA showed more accessibility in the DG. Each dot represents one DAR. The DARs shown in orange, blue and purple color match the corresponding enriched biological process terms in (**d**). **e** GO enrichment analysis of DARs with opposite accessibility changes in the DG and CA. The genes around those DARs were significantly enriched in gliogenesis and glial cell differentiation (orange), response to amphetamine (blue) and regulation of neurotransmitter levels (purple). **f** Three examples of DARs with opposite changes in accessibility in the DG and CA (**: *p* value < 0.01 calculated by edgeR; N.S., not significant *P* value > 0.05; *n* = 4 saline and 4 METH exposure samples). Those DARs were in the introns of three genes with the same expression changes in the DG and CA. **g** Genomic distribution of more and less accessible DARs in the rat genome (rn6). More than 90% of DARs were in introns and intergenic regions of the rat genome. **h** The relationship between the expression of genes associated with promoter DARs induced by METH exposure and DARs ATAC-seq signal changes in NAc and DG. **i** Distribution of rat–mouse ortholog DARs in cis-regulatory elements of the mouse genome (mm10), including CTCF-only, DNase-H3K4me3, promoter, proximal enhancer and distal enhancer. Approximately 30% of rat–mouse orthologous DARs showed enhancer function, including proximal enhancers and distal enhancers. Rat–mouse ortholog DARs: orthologous regions of rat brain DARs in the mouse genome (mm10). **j** Enriched phenotypes of rat–mouse ortholog DARs annotated with distal enhancers and proximal enhancers in the mouse. The boxplots show the median, upper and lower quartiles, maximums, and minimums.

To further explore how METH stimulus remodels the epigenetic landscape in the rat brain, we carefully examined the alteration of chromatin accessibility in all four brain regions. In total, we identified 25,598 significant differential accessible regions (DARs) in the four rat brain regions responding to METH binge; of these, 10,711 genomic loci became more accessible, and 17,816 regions became less accessible (Fig. 3a, Table 1, Supplementary Fig. 3a and Supplementary Data 6). Similar to the case for the transcriptomic changes, the DG suffered the most significant changes in chromatin accessibility among all four regions. The 12,265 regions in the DG lost chromatin accessibility after binge METH, suggesting that the DG region was the primary effect target of METH among the four regions we examined (Fig. 3a and Table 1). We further checked the expression of genes around METH exposure-induced DARs and found the genes around more accessibility DARs were generally upregulated in responding to METH exposure (Fig. 3b and Supplementary Fig. 3b, wilcox.test, *p* value < 0.05). Among DARs responding (becoming more or less accessible) to METH exposure, 89% of DARs were only identified in a single brain region, suggesting a solid region-specific epigenetic reaction to METH stimulus in the four brain regions (Fig. 3c). The GO enrichment of genes around DARs indicated distinct molecular biological processes in the four brain regions (Supplementary Fig. 3c and Supplementary Data 7). In the NAc and DG, METH exposure induced lower accessibility of genomic regions around genes enriched in neurological functions, including synaptic plasticity, neurotransmitter transport, learning, and memory.

Interestingly, the regulatory elements that gained chromatin accessibility in all four brain areas were highly enriched in nonneuronal functions. In the DG area, epigenetic changes under the METH stimulus were enriched in axon ensheathment, myelination, and oligodendrocyte and glial cell development. In the SVZ area, OCRs around immune-related genes were more open under the METH stimulus and enriched in myeloid leukocyte differentiation and endothelial cell chemotaxis (Supplementary Fig. 3b). Such evidence suggests a potential influence on glial cells after binge METH.

Similar to the oppositely-regulated DEGs between the DG and CA (Fig. 2d), such opposite regulation was also observed at the epigenetic level after the METH stimulus. Compared to two more accessible DARs and 15 less accessible DARs that were commonly shared between the DG and CA, 47% of more accessible DARs in the CA (764) were identified as less accessible DARs in the DG responding to the METH stimulus, and another 16 of less accessible DARs in the CA showed more accessibility in the DG (Fig. 3d and Supplementary Fig. 3e, f). The genes around those DARs with opposite changes in chromatin accessibility between the DG and CA were significantly enriched in gliogenesis and glial cell differentiation, response to amphetamine, and regulation of neurotransmitter levels (Fig. 3e and Supplementary Fig. 3g). We identified an enhancer located in the intron of *Tenm4*, a gene that is associated with the establishment of proper connectivity within the nervous system^63^, that exhibited significantly reduced chromatin accessibility in the DG after binge METH (Fig. 3f) and increased chromatin accessibility in the CA area, accompanied by matched gene expression changes in these two subareas of the hippocampus (Fig. 3f). Similar epigenetic changes could also be observed in the intron regions of *Etv5* and *Ptprn2* (Fig. 3f), genes that could be essential for neuronal differentiation and required for normal accumulation of neurotransmitters in the brain^64,65^.

Approximately 90% of DARs associated with METH exposure were located in intragenic or intergenic regions, except for the less accessible DARs in SVZ, suggesting that the distal regulatory elements, such as enhancers, were the primary targets that were affected by the METH stimulus (Fig. 3g, Supplementary Fig. 3h and Supplementary Data). About 5% DARs still located on the gene’s promoter regions, we found the expression of a subset of genes can positively correlate to the changed chromatin accessibility of promoter regions (Fig. 3h, Supplementary Fig. 3i). To further explore the potential functions of DARs responding to METH exposure, we first identified the orthologous regions of DARs in the mouse genome. We then checked their regulatory potential using cis-regulatory element annotation in the mouse genome from ENCODE (CTCF-only, DNase-H3K4me3, promoter, proximal enhancer, and distal enhancer)^66^. On average, nearly 40% of the mouse orthologous regions of DARs responding to binge METH stimulus are explicitly annotated as different types of cis-regulator elements in the mouse genome, and ~30% of them were annotated to have either proximal or distal enhancer function (Fig. 3i, Supplementary Fig. 3j and Supplementary Data 8), interestingly, genes associated with those two different types of enhancer DARs were enriched in distinct mouse phenotypes: genes associated with the distal enhancers were enriched in abnormal synaptic plasticity phenotypes, and genes associated with the proximal enhancers were mainly related to immunological phenotypes (Fig. 3j and Supplementary Data 9).

**Fig. 4:**
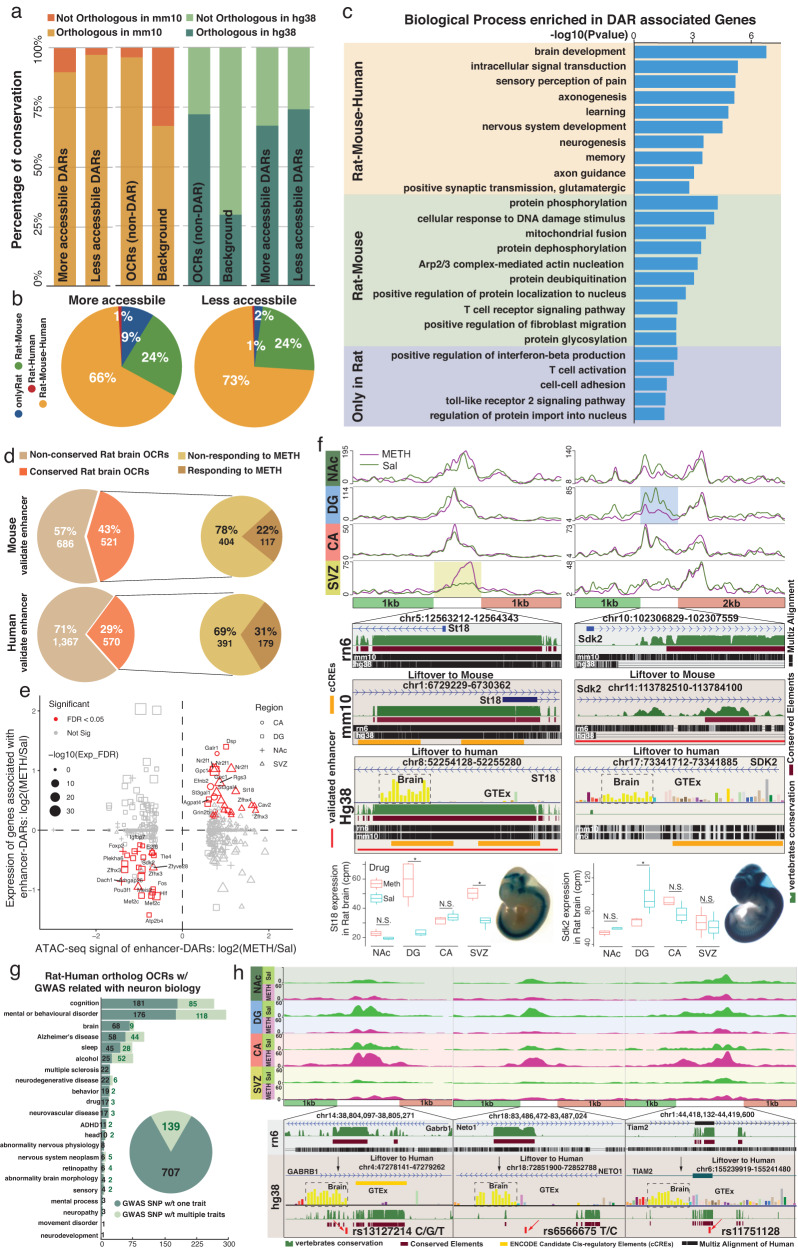
Evolutionary conservation of rat brain DARs induced by binge METH stimulus. **a** Percentages of DARs, open chromatin regions (OCRs) and background regions with orthologous counterparts in mouse and human genomes (mm10 and hg38). **b** Percentages of more and less accessible DARs with orthologs in the rat, mouse, and human genomes. Approximately 70% of DARs were conserved across the three species (rat–mouse–human DARs); 24% of DARs were rodent-specific (rat–mouse DARs), and <10% of DARs were rat-specific (rat-only DARs). **c** Biological process terms enriched in genes around (within 20 kb) DARs of three different conservation statuses: rat–mouse–human DARs, rat–mouse DARs and rat-only DARs. **d** Percentages of mouse and human-validated enhancers with ortholog sequences to OCRs in the rat brain. **e** Expression of genes associated with METH exposure-induced enhancer DARs in 4 brain regions. **f** METH exposure-induced enhancer DARs in SVZ (left) and DG (right) regions were associated with the expression changes of target genes St18 and Sdk2 (*: *p* value < 0.01, *n* = 4 saline and 4 METH exposure samples). Both enhancer DARs conserved to functionally validated orthologous in human and mouse genome. The boxplots show the median, upper and lower quartiles, maximums, and minimums. **g** Number of variants associated with neurologic phenotypes in genome-wide association studies (GWAS SNPs) that intersected with rat–human ortholog OCRs. **h** Three examples of rat–human ortholog DARs containing GWAS SNPs associated with neuron biology.

To better understand the potential biological functions of DARs responding to METH exposure, we further explored the evolutionary conservation of DARs in the human and mouse genomes. Genome-wide alignment identified that the majority of the chromatin accessible regions in the rat brain were highly conserved and had orthologous counterparts in the mouse genome (90%) and the human genome (65%), in contrast to the relatively low conservation at the genome level (70% conservation between rat and mouse genomes, 30% conservation between rat and human genomes) (Fig. 4a, Supplementary Fig. 4a-e and Supplementary Data 10). We found that ~70% of DARs associated with METH stimulus were conserved across all three species (rat–mouse–human DARs); 24% of DARs were rodent-specific (rat–mouse DARs), and less than 10% of DARs were rat-specific (rat-only DARs) (Fig. 4b). Furthermore, genes near (within 20 kb) the DARs with specific conservation statuses were enriched in specific biological process terms (Fig. 4c and Supplementary Fig. 4b, c): genes associated with the most conserved type (rat–mouse–human DARs) were highly enriched in brain function and neurological process. The genes related to rodent-specific DARs were enriched in housekeeping functions, such as protein modification, DNA damage response, and protein nuclear transportation. However, the genes associated with rat-specific DARs were more enriched in immune-related biological processes (Fig. 4c).

Since most DARs associated with the METH stimulus were far from gene promoters (Fig. 3g), we further explored the potential enhancer function of all DARs responding to binge METH exposure. Comparison with experimentally validated enhancers in mouse and human genomes^67^ indicated that 521 mouse enhancers and 570 human enhancers had orthologous sequences with rat chromatin accessible regions (Fig. 4d, Supplementary Fig. 4f, and Supplementary Data 11). Among them, 117 mouse enhancers and 179 human enhancers were evolutional conserved with DARs associated with METH stimulus (enhancer-DARs) (Fig. 4d). Although enhancers were believed to regulate target gene expression, we only found a subset of these enhancer-DARs were positively correlated with expression changes of nearby genes under METH exposure (Fig. 4e). A rat–human ortholog DAR in the promoter of the *St18* gene (rn6, chr5:12563212-12564343) was more accessible in the SVZ after METH stimulus (Fig. 4f). In the human genome, this highly conserved ortholog region (hg38, chr8:52254128-52255280) contained one validated human enhancer (hg38, chr8:52254170-52255276), which was activated in the forebrain, midbrain, and hindbrain of transgenic mice at embryonic Day 11.5 (Fig. 4f) and the human *ST18* gene is highly expressed in brain regions. Another DAR (rn6, chr10:102306829-102307559) was located in the intron of *Sdk2*, this site became more accessible in the DG after METH stimulus (Fig. 4f). The mouse ortholog region of this DAR was located in the center of one validated enhancer (mm10: chr11:113783367-113787793) that was activated in the forebrain, midbrain, and hindbrain of transgenic mice at embryonic day 11.5 (Fig. 4f).

Previous studies showed that disease-associated genetic variants were enriched in regulatory elements^68–70^. We next studied the enrichment of phenotype-associated variants from genome-wide association studies (GWAS) of diverse traits and disorders collected by GWAS Catalog^71^ in the conserved ortholog regulatory regions in the rat brain. In total, 846 SNPs associated with different neurological functions and disorders were located in 814 human–rat ortholog regions that were accessible chromatin regions in the rat brain (Fig. 4g, h, Supplementary Fig. 4g, h and Supplementary Data 12). A total of 707 SNPs were only associated with a single trait, and the remaining 139 SNPs were associated with multiple traits significantly enriched in cognition and mental/behavioral disorders. The SNP rs13127214 was associated with the unipolar depression field and was mapped to *GABRB1*, a gene related to inhibitory synaptic transmission in the vertebrate brain^72^. SNP rs13127214, located in the human–rat conserved region (rn6, chr14:38,804,097-38,805,271), became less open in the DG but more open in the CA and SVZ after METH stimulus (Fig. 4h). We also found that the intronic SNP rs6566675 in *NETO1*, which plays critical roles in spatial learning and memory^73,74^, is located in the DAR chr18:83,486,472-83,487,024, which is less open in the NAc and DG but more opened in the CA after METH stimulus (Fig. 4h). The DAR chr1:44,418,132-44,419,600, less open in the NAc and DG but more open in the CA, overlapped with the exon of the *Tiam2* gene (Fig. 4h). After alignment to the human genome, an orthologous region (chr6:155,239,919-155,241,480) was located within the *TIAM2* gene, which is highly expressed in the human brain and may play a role in the neural cell development^75,76^. This conserved regulatory region contained a GWAS SNP (rs11751128) associated with human intelligence^73^.

### METH exposure induced a distinct gene regulatory network in the rat brain

**Fig. 5:**
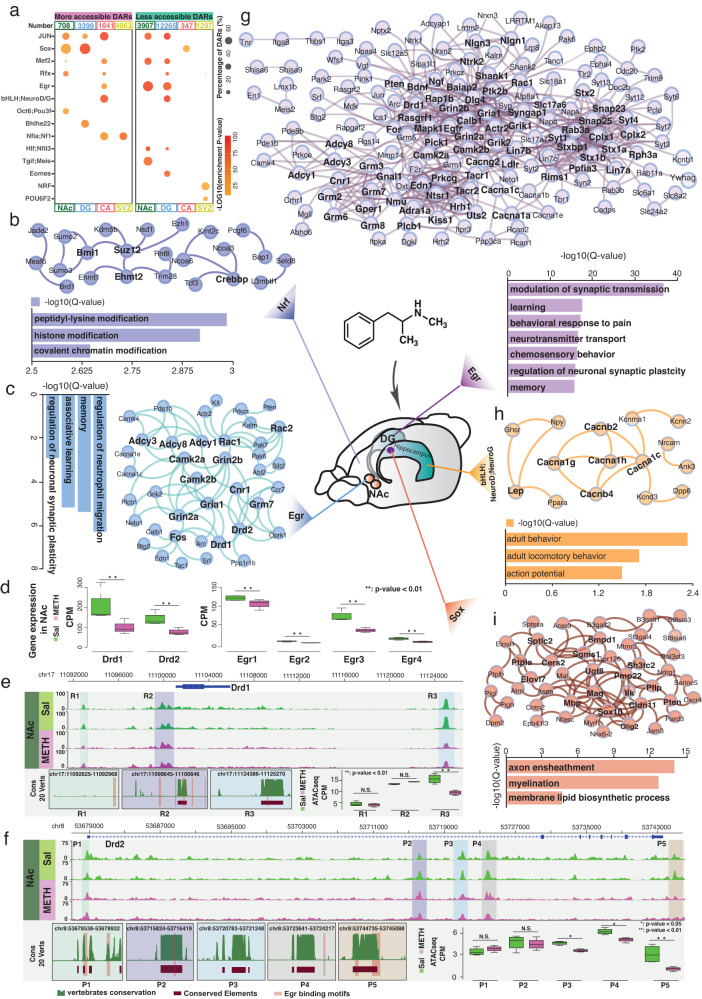
Distinct gene regulatory networks in 4 rat brain regions responding to binge METH exposure. **a** Transcription factors binding motifs enriched in more and less accessible DARs of 4 rat brain regions in response to binge METH exposure. The size of the circle represents the percentage of DARs containing TFs binding motifs. The color scale represents the enrichment *p* value. **b** Enriched biological process terms of DARs with Nrf binding motifs in the SVZ and the gene regulatory network built by DAR-associated genes. **c** Enriched biological process terms of DARs with Egr binding motifs in the NAc and the gene regulatory network built by DAR-associated genes. **d** Gene expression of *Drd1/2* and *Egr* family genes in the NAc in response to METH exposure (**: *p* value < 0.01, *n* = 4 saline and 4 METH exposure samples). The OCRs around *Drd1* (**e**) and *Drd2* (**f**) in the NAc with the following analysis results: 20-vertebrate conservation of OCR, *Egr* binding motifs in OCR and open chromatin signals of OCR in METH exposed and Sal samples (**: *p* value < 0.01, N.S., not significant *P* value > 0.05, *n* = 4 saline and 4 METH exposure samples). The boxplots show the median, upper and lower quartiles, maximums, and minimums. Enriched biological process terms and gene regulatory networks for DARs with *Egr* binding motifs in the DG (**g**), DARs with bHLH/NeuroD/NeuroG binding motifs in CA (**h**), and DARs with *Sox* binding motifs in the DG (**i**).

To further explore the molecular regulation underlying the DAR responses to binge METH stimulus, we performed transcription factor binding motif enrichment analysis for more and less accessible DARs in four rat brain regions. The binding motifs of many neuronal function-related TFs, including *Sox*, *Egr*, and *NeuronD1*, were highly enriched in the DARs associated with METH stimulus (Fig. 5a, Supplementary Fig. 5a-c and Supplementary Data 13, 14). In SVZ regions, we noticed *Nrf* motif was highly enriched in the more accessible DARs induced by METH exposure. The protein–protein interaction (PPI) network of genes around these DARs was associated with histone modification (Fig. 5b), including the major components of the polycomb group complex 1, such as *Suz12*, *Ehmt2*, and Bmi1. Such results suggested the remodeling of histone repressive methylation might be corporate with chromatin accessibility changes after METH exposure.

We found 2434 (62.3%) less-accessible DARs in the NAc containing *Egr* binding sites, and genes around these *Egr* binding DARs were significantly enriched in neurological biology terms, such as memory, learning, and synaptic plasticity (Fig. 5c and Supplementary Fig. 5b). We built a PPI network for the genes in these enriched biological process terms in the NAc (Fig. 5c and Supplementary Data 15). In the PPI network, *Rac1, Rac2*, *Drd1*, *Drd2*, *Camk2a, Camk2b*, *Gria1*, *Grin2a*, and *Fos* were found to be highly connected to other *Egr* targets, suggesting that *Egr* might regulate these network hubs in response to binge METH stimuli in the NAc region. *Rac1* is well known to be associated with cocaine addictive behavior^77,78^, and *Camk2a* is considered to be involved in the loss of control of ethanol consumption and cocaine dependence^79,80^. *Gria1*, *Grin2a*, and *Fos* are highly implicated in drug addiction behavior and synaptic plasticity^81–84^.

We specifically focused on the *Drd1* and *Drd2* genes, which are critical dopamine receptors in the NAc region and play central roles in responding to addictive behaviors for most drugs^85^. After the METH stimulus, we noticed that the downregulation of *Drd1* and *Drd2* was highly correlated with reduced expression of the *Egr* family (Fig. 5d and Supplementary Fig. 5d). Three open chromatin regions around *Drd1*, including the promoter and potential enhancer regions, all contain *Egr* binding sites (Fig. 5e). We found that the 3′ downstream enhancer OCR (R3) lost open chromatin signals significantly after METH stimulus. The *EGR* binding motif in this METH exposure-associated OCR was conserved between humans and rats (Fig. 5e). Similarly, three (P3, P4, and P5) of five OCRs around the *Drd2* gene significantly lost open chromatin signals after the binge METH stimulus. All five OCRs contained conserved elements between the human and rat genomes and harbored *Egr* binding motifs (Fig. 5f). Such results suggested that *Egr* might regulate *Drd1* and *Drd2* with a similar mechanism in both rat and human brains.

In the DG region, 5088 DARs were found to contain the *Egr* binding motif, which accounted for 41.5% of the total open chromatin regions that lost open chromatin signals after the METH stimulus (Fig. 5a, Supplementary Fig. 5a and Supplementary Data 14). Genes around these less opened DARs were highly enriched in synaptic transmission, learning, and behavioral response to pain (Fig. 5g, Supplementary Fig. 5b, e, and Supplementary Data 15). In the PPI network of these *Egr* targets in the DG region, the top key nodes tightly associated with distinct psychiatric disorders and addictive behaviors in previous studies included *Syt1*, *Stx1a*, *Dlg4*, *Cplx1*, *Gria1*, *Snap25*, *Rab3a*, and *Bdnf*^81,86–91^. Our results suggested that these essential genes could all be regulated by the *Egr* family and emphasize the importance of *Egr* in the rat DG region in response to METH stimulus.

In the CA regions, 648 DARs were found to contain the *NeuronD1(bHLH)* binding motif, which accounted for 39.5% of the total open chromatin regions that gain open chromatin signals after the METH stimulus (Fig. 5a, Supplementary Fig. 5a and Supplementary Data 14). These *NeuronD1(bHLH)* enriched METH exposure-induced DARs located around the genes associated with action and locomotory behaviors, such as Calcium Voltage-Gated Channel Subunit Alpha1family members, including *Cacna1c*, *Cacna1g*, *Cacna1h*, and other channel auxiliary subunits, *Cacnb2* and *Cacnb4* (Fig. 5h). Many of these genes were found to associate with neurodevelopmental disorders, autism spectrum disorders, psychiatric disorders, seizures, epilepsy, and episodic ataxia^92–98^.

Meanwhile, we found that the Sox binding motif was highly enriched in the METH exposure-induced more-open DARs in both the NAc and DG regions (Fig. 5a). A total of 62.4% of NAc more-open DARs (442) and 70% of DG more-open DARs (2,380) contained *Sox* binding motifs (Supplementary Fig. 5a and Supplementary Data 14). The genes around these *Sox* binding DARs in the DG region were highly enriched in axon ensheathment, myelination, and membrane lipid biosynthetic processes (Fig. 5i, Supplementary Fig. 5b, f, and Supplementary Data 15). PPI network analysis suggested that *Mbp*, *Sox10*, *Mag*, *Cldn11*, *Ugt8*, *Pmp22*, and *Cers2* were the top connected nodes with critical regulatory roles in myelination and lipid metabolism and ensheathment in the oligodendrocytes^99–102^.

## Discussion

Methamphetamine (METH) is a stimulant amphetamine drug that is extremely addictive, with 61% of individuals treated for METH use disorder relapsing within 1 year^15–17^. Recently, developed animal models, especially rodent models, helped us better understand the molecular consequences of substance misuse^1,22,31^. Although previous studies illustrated that METH exposure could cause dramatic epigenetic changes in the NAc and frontal cortex^1,3,31,37,103^, there is still a lack of a clear description of the molecular changes in different brain regions under exposure to neurotoxic METH doses.

Here, we explored the METH-induced epigenetic and transcriptomic changes in four areas of rat brains, namely the NAc, DG, CA, and SVZ. This work represents the most comprehensive dataset to date of METH-induced transcriptome and chromatin accessibility from multiple rat brain regions. In our study, genome-wide epigenetic alterations and dynamic gene expression changes induced by acute binge METH exposure were found in all four brain regions, indicating the strong molecular response in the whole brain to the neurotoxic METH exposure. More importantly, the molecular changes induced by METH were rarely shared among different areas, suggesting that different brain regions respond to METH exposure in a highly region-specific fashion. For example, approximately one thousand genes underwent expression changes in response to METH exposure in the DG and SVZ regions, while approximately three hundred genes underwent expression changes in the NAc and CA regions (Fig. 2a). Moreover, unlike the region-specific DEGs, the expression changes of METH exposure-induced DEGs were  not well correlated to the chromatin accessibility change on the promoter region. Recent studies^24,104,105^ suggested that many genes can be regulated independently from the changes in chromatin accessibility. All these results suggested more complex regulatory mechanisms existed besides epigenetic remodeling, like pause-release^106^, enhancer RNA regulation^107^, miRNA interference^108^, mRNA stability^109^, etc. Meanwhile, a relatively stable epigenetic landscape on gene promoter might work as epigenetic memory and could be a feasible way to allow gene expression back to normal after being rapidly regulated by other mechanisms under METH exposure. We also noticed substantial expression alterations in many genes associated with histone modification and chromatin remodeling after METH exposure in all four brain regions (Fig. 2f, g). This finding provides evidence that complicated epigenetic remodeling events at different levels can be induced by METH exposure, as previous studies have reported^1,3,18,23,31,37,110^.

Previous studies suggested METH has different toxic effects in these four brain regions examined in this study, and the CA was the most affected while the other three regions were much less affected by measuring the neurogenesis and neuroplasticity markers^54,111–113^. However, we noticed a more significant molecular response to toxic METH exposure in neurogenic SVZ and DG regions, when compared to non-neurogenic CA and NAc regions. Moreover, we also noticed that two subregions of the hippocampus, CA and DG, showed the opposite responses to METH exposure at both the transcriptomic (Fig. 2d) and epigenetic (Fig. 3d) levels. These oppositely regulated genes were highly associated with responses to different substances in the brain, memory, neuron, and glial cell differentiation (Fig. 2e, Fig. 3e). The opposite response between the CA and DG might be associated with the specific biological functionality and neuron maturity of the two subregions. As a part of the hippocampal formation in the temporal lobe of the brain, the DG is generally believed to contribute to the formation of new synapse connections and episodic memories^57,114–116^. The CA region of the hippocampus plays an important role in long-term memory^57,58^. Thus, even short-term METH stimulation might create new synaptic connections in the DG region, affecting the long-term memory in the CA region. Moreover, in the DG region, we noticed that the open chromatin regions (OCRs) around genes associated with glial cell differentiation were less open after METH exposure (Fig. 3e), and the same OCRs became more open in the CA. This result suggested that the glial cells in the hippocampus might be more active and vulnerable to METH exposure. Moreover, we noticed the expression of genes response to cytokine were upregulated in the CA, suggesting that the CA might have specific stress recovery mechanisms from METH exposure, since the inflammation could be a protective response preparing the tissue for repair and healing after neurotoxic damage.

We also performed a comparative genome analysis to understand the potential function of DARs identified in different brain regions. Over 70% of DARs associated with METH exposure were conserved between humans and rats. The genes around these DARs were highly enriched in fundamental functions of the brain, such as brain development, neurogenesis, learning, and memory (Fig. 4a, c). In contrast, genes around rodent- and rat-specific DARs were more often associated with protein modification and immune response; such results emphasize the unique species-specific characteristics of the rat model in addiction research. We also noticed many known SNPs associated with neuronal biology and disease located in the conserved human ortholog counterparts of DARs responding to METH exposure in the rat brain (Fig. 4g, h). Most importantly, we found that more than 300 orthologous of these DARs had the validated brain-specific activation (Fig. 4d, e, f). Thus, we firmly believe that the conserved human orthologs of DARs identified in this study are highly likely to play essential roles in METH-induced toxicity in the human brain.

We also constructed regional-specific regulatory networks in response to METH exposure in the rat brain (Fig. 5b, c, g, h, i). By using a genomic DNA context-based approach, we were able to connect crucial upstream TFs regulators to their downstream target genes and eventually better understand the molecular response to METH exposure at an upstream regulatory level. For example, both *Drd1* and *Drd2* are critical players in the reward pathway directly associated with addiction to multiple substances, including METH^85^. Meanwhile, the *Egr* family is broadly affected by METH exposure^110,117–119^. Our results provide information regarding the changes in *Drd1/2* expression in response to METH exposure in the rat NAc region through the *Egr* family. Meanwhile, certain evidence indicates that METH exposure greatly affects the biological functions of glial cells^120–122^. In both the NAc and DG regions, our results indicated that *Sox* binding sites were highly enriched in the DARs associated with genes that regulate myelination and ensheathment, suggesting that *Sox* family members could be upstream regulators in glial cells responding to METH exposure.

There are still certain limitations in our study. First, this study only included male rats, thus the identified transcriptomic and epigenetic changes in responding to binge METH exposure might differ in female animals. Second, although the ATAC-seq method we used in this study supplied a high-resolution open chromatin landscape under METH exposure, we still lack an understanding of other epigenetic changes in responding to binge METH exposure, such as DNA methylation and different histone modifications. Finally, as the most comprehensive study so far, there were still only four important brain regions included in the current study, and more research should be performed to cover other important brain regions in responding to addictive substances.

In general, our study emphasized the importance of applying multi-omics approaches in addiction research, and our results can serve as the basis for further studies.

## Methods

### Animals

Adult male Sprague–Dawley rats (Harlan, Indianapolis, IN, USA) (weighing 250–300 g on arrival) were pair-housed under a 12 h light/dark cycle in a temperature-controlled (20–22 °C) and humidity-controlled room. Food and water were available ad libitum. The animals were allowed to acclimatize for 1 week before the start of the study. All animal procedures were conducted between 7:00 A.M. and 7:00 P.M. in strict accordance with the National Institutes of Health (NIH) Guide for the Care and Use of Laboratory Animals and were approved by the Institutional Animal Care and Use Committee (IACUC) at Wayne State University. The description of animal procedures meets the ARRIVE recommended guidelines described by The National Centre for the Replacement, Refinement and Reduction of Animals in Research^123^.

### Methamphetamine administration

( + )-Methamphetamine hydrochloride (METH, 10 mg/kg free base) (Sigma-Aldrich, St. Louis, MO) or saline (1 mL/kg) was administered to the rats every 2 h in four successive intraperitoneal (i.p.) injections, as previous studies^124–126^. To measure hyperthermia, the core body temperatures of the rats were measured with a rectal probe digital thermometer (Thermalert TH-8; Physitemp Instruments, Clifton, NJ) before the beginning of the treatment (baseline temperatures) and at 1 h after each METH or saline injection. All METH-treated rats in this study reached 39 °C indicating neurotoxicity (Supplementary Fig. 6a). Rats were sacrificed by decapitation at 24 h after the last injection of the drug or saline.

### Tissue collection and storage

The brains were removed and placed in the rat brain metal dye pre-chilled on crushed ice. A coronal section between −2mm to −3mm AP (anterior-posterior) was made, creating a division of each brain into two main sections: an anterior section and a posterior section, as shown in Supplementary Fig. 6b-d. The anterior section containing the NAc and SVZ was sliced into 2mm-thick brain slices. The slices that contain the NAc and SVZ were removed and frozen on top of the dry ice. Subsequently, the NAc and SVZ were punched out using a 1 mm tissue puncher. The posterior part of the brain was placed on an inverted glass beaker that was pre-chilled with crushed ice and divided into posterior hemispheres. After the top neocortex/corpus callosum layer was removed using fine dissection tools, the DG and CA were scooped out. After the dissection, tissue pieces were immediately fast-frozen and stored at −80 °C until shipped on dry ice for analyses. Two hemispheres of the same rat brain were collected in this study, one hemisphere was used to perform RNA-seq and another one was used for ATAC-seq.

### Library construction

Total RNA was isolated via TRIzol Reagent (Thermo Fisher Scientific, 15596026), Phasemaker Tubes (Thermo Fisher Scientific, A33248) and RNA Clean & Concentrator-5 (Zymo Research, R1013). In brief, rat brain tissues were homogenized in 1 ml of TRIzol Reagent, the tissue lysates were transferred to a pre-spined Phasemaker Tube. 0.2 ml of chloroform was added to the tube, the tubes were then shaken for 15 s. The mixture was centrifuged at 16,000 *g* for 5 min, the top aqueous phase was transferred to a microcentrifuge tube. An equal volume of ethanol was added to the aqueous phase. The RNA purification with DNase treatment was performed following the manual of the RNA Clean & Concentrator kit. Then the Ribosomal RNAs were removed from 500 ng for the total RNA using the NEBNext rRNA Depletion kit (NEB, E6310). Skipping the mRNA isolation part, RNA-seq libraries were then constructed using 10 ng of rRNA-depleted total RNA with Universal Plus mRNA-seq kit (TECAN, 0520-A01) following the kit manual. 2 × 75 bp paired-end sequencing was run for all libraries on the Illumina NextSeq 500 platform.

ATAC-seq was generated using the omni ATAC-seq protocol for frozen tissues (Nature Methods volume 14, pages 959–962, 2017). In brief, rat brain tissues were homogenized in 2 ml of cold 1× homogenization buffer. Nuclei were layered from the tissue lysate with iodixanol solution. 50,000 nuclei were used in the transposition reaction with 100 nM of Transposase (Illumina, 20034197). The ATAC-seq libraries were prepared by amplifying for 9 cycles on a PCR machine with NEBNext High-Fidelity 2× PCR master mix (NEB, M0541). 2 × 75bp paired-end sequencing was run for all libraries on the Illumina NextSeq 500 platform.

### Raw sequence data and processing

Total 32 RNA-seq fastq files and 29 ATAC-seq fastq files were generated from 4 brain regions, including Nucleus accumbens (NAc), Dentate gyrus (DG), Ammon’s horn (CA), and Subventricular zone (SVZ). For both RNA-seq and ATAC-seq, each region had 4 samples with saline treatment (Sal) and 4 samples with methamphetamine binge (METH binge), except SVZ region only had 3 Sal samples and 2 METH binge samples for ATAC-seq data.

ATAC-seq data of 4 brain regions were separately processed by AIAP package that contained an optimized ATAC-seq data QC and analysis pipeline with default parameters^127^. Open chromatin regions (OCR) generated by AIAP were used in downstream analysis. Then, mergeBed was used to generate consensus OCRs of two conditions of 4 brain regions^128^.

RNA-seq data were processed as in previous studies^129^. RNA-seq data of 4 rat brain regions were processed by Cutadapt (v2.7; --quality-cutoff=15,10 --minimum-length=36), FastQC (v0.11.4), and STAR (v2.5.2b; --quantMode TranscriptomeSAM --outWigType bedGraph --outWigNorm RPM) to do the trimming, QC report and rat genome mapping (rn6)^130–132^. Then, gene expressions in rat brain regions were calculated by featureCounts (-p -T 4 -Q 10) based on UCSC gene annotation of rat^133,134^.

Both RNA-seq and ATAC-seq data were normalized by following the manual of the RUVSeq package as in previous studies^135,136^. In general, the gene counts and ATAC peak counts were first upper-quartile normalized (edgeR), and factor analysis was performed by using the residuals calculation of RUVr function (RUVSeq). The variations within the dataset that were not correlated to tissue and Meth exposure, including bias of tissue dissection and library sequencing, were further removed by using a general linear regression model fitting process.

### Region-specific genes and OCRs in 4 regions of normal male rat brain

EdgeR was used to identify region-specific genes that were significantly highly expressed in one normal region compared to the other three regions with the cutoff log2FoldChange>log2(2) and FDR < 0.01 to ensure the sensitivity and specificity^136,137^. The GO enrichment analysis of region-specific genes in 4 regions was performed by DAVID (Database for Annotation, Visualization, and Integrated Discovery, v6.8)^138,139^. The region-specific OCRs were also identified in 4 rat brain regions with EdgeR method with the cutoff log2FoldChange>log2(2) and FDR < 0.01 to ensure the sensitivity and specificity^137^. Then, the average signals of normal ATAC-seq samples in each region were generated by bigWigMerge and visualized by plotHeatmap of deepTools software (v3.5.0)^140^. The mouse ortholog regions of region-specific OCRs were generated by liftOver with parameter “-minMatch=0.6” and then those ortholog regions were used to identify enriched biological process terms in 4 brain region with GREAT (version 4.0.0)^134,141^. The analysis settings of GREAT included that: (1) Species assembly: Mouse, NCBI build 38; (2) Background regions: whole-genome; (3) Association rule: Basal plus extension. Then, top 20 enriched terms of biological process in 4 rat brain regions were filtered with cutoffs of Binom FDR Q-Val < 0.05 and Hyper FDR Q-Val < 0.05 simultaneously. The correlation between expression of region-specific genes and those genes’ promoter ATAC-seq signal was calculated by ‘cor’ function of R package (version 4.3) with pearson method.

### Binge METH exposure induced differential expression genes in 4 male rat brain regions

The significantly differential expression genes (DEGs) induced by METH binge in 4 rat brain regions vs. saline samples were identified by EdgeR method with the cutoffs of log2FoldChange>log2(2) and FDR < 0.01 to ensure the sensitivity and specificity. The Venn plots of intersection for up- and down-regulated DEG among 4 regions were respectively generated by jvenn^142^. The enriched terms of the biological process were identified by DAVID separately for up- and down-regulated DEG. Next, the gene lists of transcription factors and epigenetic modification factors (Epi-Modifiers) were separately gathered from the AnimalTFDB3.0 and Epi-Modifiers databases^143,144^.

### Identification of METH exposure-induced differential accessible regions in 4 rat brain regions

The EdgeR was used to identify significantly differential accessible regions (DARs) induced by METH binge in 4 brain regions compared to saline samples with a stringent cutoff of log2FoldChange > log2(1.5) and FDR < 0.001 to ensure the optimized sensitivity and specificity, as the previous study suggested^137^. The log2 values of the differential accessibility in 4 brain regions after METH binge stimulus were generated based on bigwig files by bigwigCompare and visualized by plotHeatmap of deepTools^140^. The Venn plots of intersection for both more and less accessible DARs among 4 brain regions were respectively generated by jvenn^142^. The liftOver was used to identify ortholog regions of rat brain DARs in the mouse genome (mm10) with parameter “-minMatch=0.6”^134^. Then, those ortholog regions were used to identify the top 20 enriched terms of biology process in 4 rat brain regions by GREAT with default parameter and cutoffs of Binom FDR Q-Val < 0.05 and Hyper FDR Q-Val < 0.05^141^. Three examples of DARs with reversed accessibility between DG and CA were visualized by WashU Epigenome Browser. The intersectBed method was used to determine the number of DARs across different genomic features (promoters, exons, introns, and intergenic regions), which were defined by using the UCSC gene annotation of the rat genome. The mouse ortholog regions of rat brain DARs intersected with mouse cis-regulation elements (CREs) from ENCODE database were used to explore the potential regulatory function of those DARs^66^. Then, GREAT was used to identify enriched mouse phenotypes of those ortholog DARs with different CRE annotation types.

### Evolutionary conservation of DARs

The orthologous regions in mouse (mm10) and human (hg38) genomes for OCRs in rat brain regions were separately identified by using liftOver software with parameter “-minMatch=0.6” [65]. The rat genome was divided into 500 bp windows as background regions and the orthologous conservation of background regions were measured as the same standard as OCRs described above. The rat-brain DARs were classified into 4 groups based on the ortholog in mouse and human genomes: Rat-Mouse-Human DARs were conserved across rat, mouse and human genomes; Rat-Mouse or Rat-Human DARs were DARs of rat had orthologous regions only in mouse or human genome; onlyRat DARs were not orthologous in mouse and human genomes. Then, those DARs form different groups were mapped to nearest genes within 20 kb away, and the specific DAR-mapped genes of those 4 groups were separately used to identify the enriched terms of the biological process by DAVID.

The experimentally validated human (hg19) and mouse (mm9) enhancers were downloaded from VISTA Enhancer Browser database and were separately transferred to the coordinates in hg38 and mm10 genomes by liftOver with parameter ““-minMatch=0.95”^67,134^. Then, intersectBed method was used to separately identify the mouse and human-validated enhancers that can be overlapped by mouse and human orthologous regions of rat brain OCRs. The two examples of DARs that had orthologous intersections with human and mouse-validated enhancers were visualized by WashU Epigenome Browser^145^. The results of vertebrates-conservation, multiz-alignments, CREs, and gene expression in the GTEx data portal were generated by USCS genome browser^134^. The activation patterns of those two validated enhancers at E11.5 stage were also downloaded from VISTA Enhancer Browser.

The variants-trait information of genome-wide association studies (GWAS) were downloaded from GWAS Catalog (https://www.ebi.ac.uk/gwas/home)^71^, there were a total 113,705 GWAS SNPs associated with different traits. The GWAS SNPs associated with the neurological process were identified based on mapped traits. Then, intersectBed was used to overlap those GWAS SNPs and rat-human orthologous OCRs. Three examples of rat-human orthologous DARs containing GWAS SNPs were visualized by WashU Epigenome Browser. Visualization of vertebrates-conservation, alignment, CREs, and GTEx expression associated with OCRs was generated by using the USCS genome browser.

### Motif enrichment of DAR and Gene regulation network

The transcription factors binding motifs (TFBS) enriched in more and less accessible DARs of 4 rat brain regions were separately analyzed by using findMotifsGenome.pl (-size given) of HOMER software (v4.11.1)^146^. The significantly enriched de novo binding motifs in 4 regions were identified with three conditions: (1) at least 5% of accessible DARs in one region contained the TFBS; (2) the match score of TFBS should be >0.85; (3) *P* value of TFBS should be <1e−11. Then, known transcription factors under the enriched TFBS were extracted with a match score > 0.85.

The DARs containing the Egr, Sox, and NeuroD1 binding motifs were extracted from the HOMER results and ortholog regions in the mouse genome of those DARs were used to identify enriched biological process terms with GREAT. The DARs-associated genes in the neurological biological process were extracted and used to build the gene regulation networks by using String^147^ database.

The open chromatin signals of OCRs around Drd1 and Drd2 genes in NAc region were visualized by WashU Epigenome Browser. The vertebrates-conservation of OCRs associated with Drd1 and Drd2 genes were generated by the USCS genome browser. The FIMO software was used to scan TFBS motifs in those OCRs associated with Drd1 and Drd2 genes based on the motif weigh matrix file (JASPAR_CORE_2016_vertebrates.meme) from JASPAR^148,149^.

### Statistics and reproducibility

All computational analyses have been performed using R package (version 4.3) and EdgeR package was used to identify the differential expressed genes and open chromatin regions between METH exposure and saline samples and to calculate the statistic difference, unless otherwise noted. The correlation values were calculated by cor function of R with pearson method. The reads count of genes and OCRs were normalized by using RUVseq package of RUVr method. The pheatmap function in R and deepTools were separately used to generate heatmaps for RNA-seq and ATAC-seq data. The DAVID and GRETA methods were separately used to perform GO-term enrichment analysis for DEGs and DARs. All the details are provided in the Methods section.

The stringent statistical cutoffs were applied to identify DEGs (log2FoldChange > 1 and FDR < 0.01) and DARs (log2FoldChange > log2(1.5) and FDR < 0.001). The multiple comparisons were corrected and controlled by using the FDR method. Our previous study showed that the signal distribution of ATAC-seq data was distinct from that of RNA-seq data^136^. Then, the thresholds for DAR analysis in the current manuscript are the optimized parameters in our comprehensive benchmarking study that allow a well-balanced sensitivity and specificity in DAR identification^136^.

There were 32 RNA-seq, and 29 ATAC-seq data from 4 brain regions of male rat. For RNA-seq data, NAc, DG, CA, and SVZ regions all had 4 replicates of saline control and 4 replicates under METH exposure and were sequenced in 2 different batches; For ATAC-seq data, except 3 Sal samples and 2 METH binge samples, the other 3 regions had the same replicates number as RNA-seq.

### Reporting summary

Further information on research design is available in the Nature Portfolio Reporting Summary linked to this article.

### Supplementary information

Supplementary information

Description of Additional Supplementary Files

Supplementary Data 1

Supplementary Data 2

Supplementary Data 3

Supplementary Data 4

Supplementary Data 5

Supplementary Data 6

Supplementary Data 7

Supplementary Data 8

Supplementary Data 9

Supplementary Data 10

Supplementary Data 11

Supplementary Data 12

Supplementary Data 13

Supplementary Data 14

Supplementary Data 15

Reporting Summary

## Data Availability

All sequencing data in this paper has been deposited through Gene Expression Omnibus (GEO) repository: GSE199374. The variants-trait information of genome-wide association studies (GWAS) were downloaded from GWAS Catalog (https://www.ebi.ac.uk/gwas/home). the gene lists of transcription factors and epigenetic modification factors (Epi-Modifiers) were separately gathered from the AnimalTFDB3.0 and Epi-Modifiers databases. The experimentally validated human (hg19) and mouse (mm9) enhancers were downloaded from VISTA Enhancer Browser database. The source data were deposited to FigShare: Fig. 110.6084/m9.figshare.24070365.v1, Fig. 210.6084/m9.figshare.24070482.v1, Fig. 310.6084/m9.figshare.24070527.v1, Fig. 410.6084/m9.figshare.24070593.v1, Fig. 510.6084/m9.figshare.24070608.v1, Supplementary Fig. 110.6084/m9.figshare.24070635.v1 Supplementary Fig. 210.6084/m9.figshare.24070662.v1 Supplementary Fig. 310.6084/m9.figshare.24070722.v, Supplementary Fig. 4 10.6084/m9.figshare.24070776.v1, and Supplementary Fig. 5 10.6084/m9.figshare.24070800.v1.

## Code availability

All codes and source data used for the analysis and plots generated in this study were available here^150^: https://github.com/Zhang-lab/Rat_Brain_Meth.git; 10.5281/zenodo.8170364.
