# Supplementary material for: Methamphetamine-induced region-specific transcriptomic and epigenetic changes in the brain of male rats
Source: Commun Biol. 2023 Sep 27;6:991. doi: 10.1038/s42003-023-05355-3 (PMC10533900; doi:10.1038/s42003-023-05355-3)
Supplement: Supplementary file 1 — Supplementary information [file 42003_2023_5355_MOESM1_ESM.pdf]

SUPPLEMENTARY INFORMATION

Methamphetamine-induced regional-specific transcriptomic and epigenetic changes in the brain of male rats

Benpeng Miao[1,2], Xiaoyun Xing[2], Viktoriia Bazylianska[3], Pamela Madden[4], Anna Moszczynska[3*], Bo Zhang[1*]

1. Department of Developmental Biology, Center of Regenerative Medicine, Washington University School of Medicine, St. Louis, MO 63110, USA
2. Department of Genetics, Center for Genomic Sciences and Systems Biology, Washington University School of Medicine, St. Louis, MO 63110, USA
3. Department of Pharmaceutical Sciences, Wayne State University, Detroit, MI, 48201, USA
4. Department of Psychiatry, Washington University School of Medicine, St. Louis, MO 63110, USA

* Corresponding:

Anna Moszczynska: ei2744@wayne.edu

Bo Zhang: bzhang29@wustl.edu

Supplementary figures: 6

Supplementary Figures

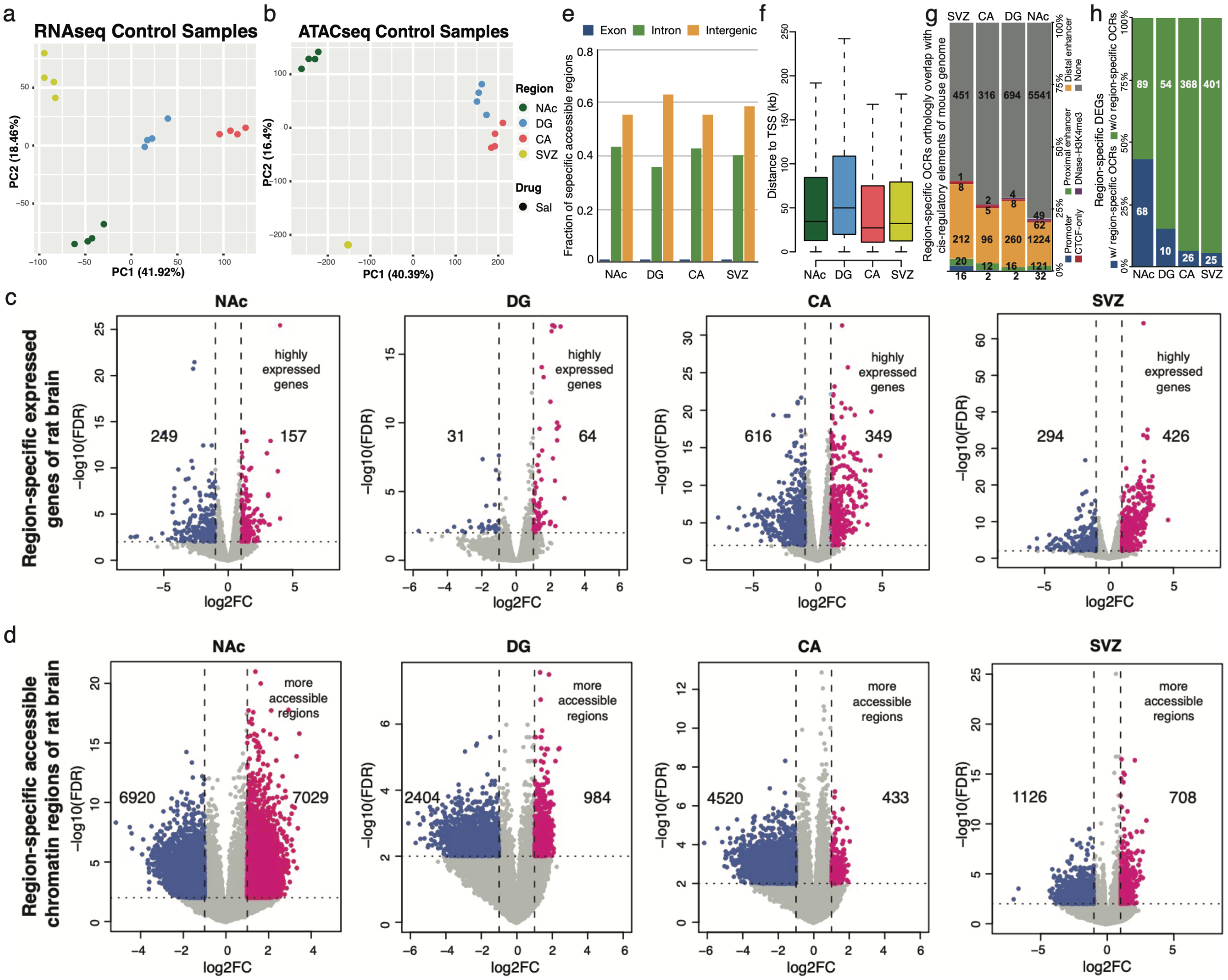

Supplementary Figure 1. RNA-seq and ATAC-seq data of 4 brain regions from control and binge METH exposure rat samples. The principal analysis (PCA) of RNA-seq data (**a**) and ATAC-seq data (**b**) from 4 normal rat brain regions. **c-d**, The volcano plots of region-specific expressed genes (**c**) and accessible chromatin regions (**d**) in 4 rat brain regions. Red dots represented the specific genes and accessible regions in each brain region. Log2FC: log2 value of fold changes with one normal region against other regions; -log10(FDR): -log10 value of false discover rate (FDR). **e**, Genomic distribution of 4 rat brain region-specific accessible regions in rat genome: exon, intron, and intergenic regions. Large fraction of those specific accessible regions located in nitrogenic and intergenic regions. **f**, Boxplot about the distance to TSS of region-specific accessible regions in 4 rat brain regions. The boxplots show the median, upper and lower quartiles, maximums, and minimums. **g**, Distribution of rat–mouse ortholog region-specific OCRs in cis-regulatory elements of the mouse genome (mm10), including CTCF-only, DNase-H3K4me3, promoter, proximal enhancer and distal enhancer. **h**, The percentage of region-specific DEGs around region-specific OCRs in 4 brain regions.

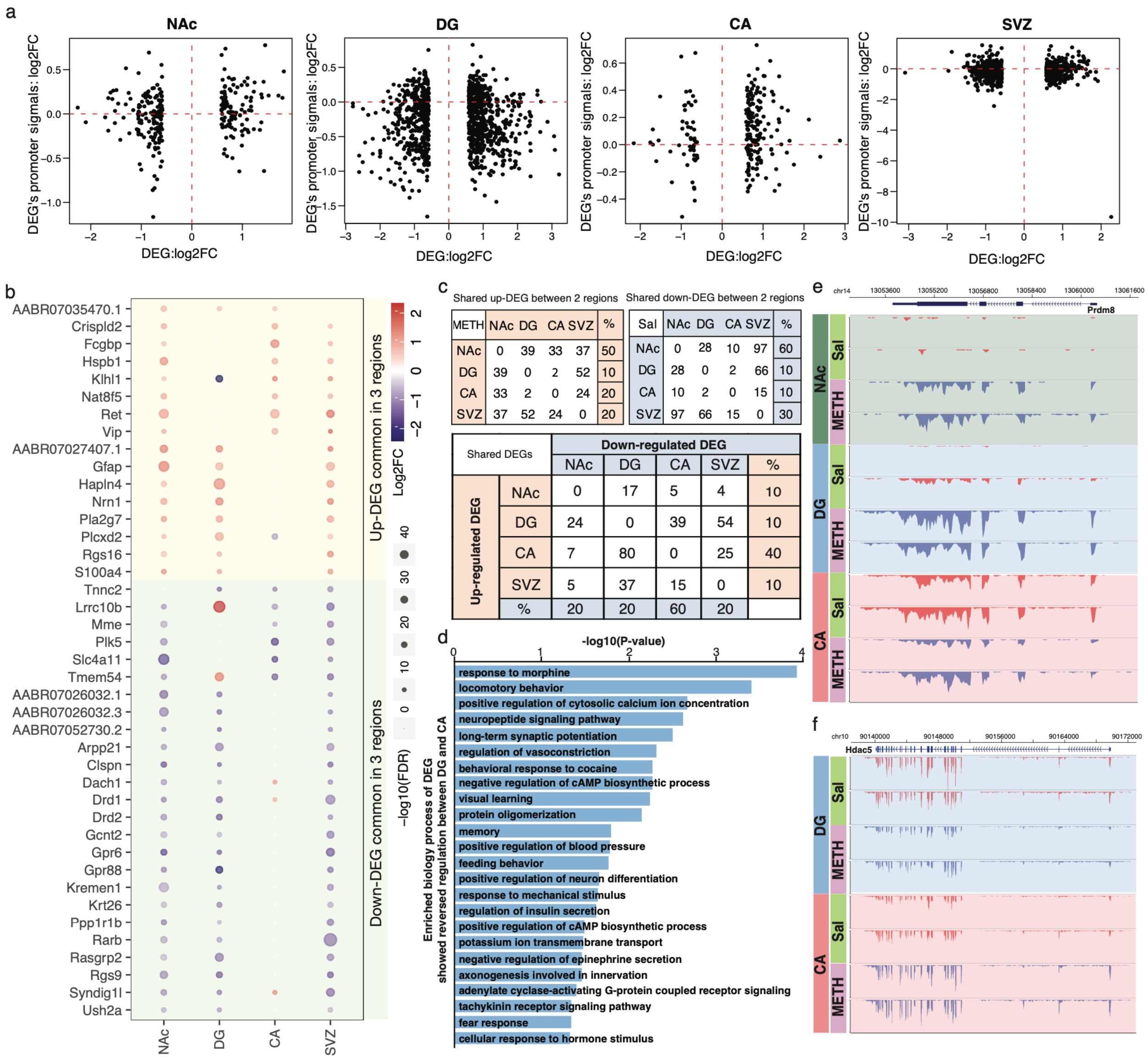

Supplementary Figure 2. The differential expressed genes (DEGs) identified in 4 rat brain regions responding to binge METH exposure. a, Correlation between ATAC-seq signal on promoters and expression of METH-induced DEGs in all four brain regions. **b**, Up and down-regulated DEGs were common in at least 3 brain regions. **c**, Number of common genes between 2 regions separately for up and down-regulated DEGs, and the number of common DEGs between DG and CA showed reversed regulation pattern. Large percent of up and down-regulated DEGs in NAc were also identified in another one region. And large percent of DEG in CA regions showed reversed regulation pattern in another one region, especially in DG region. **d**, Biology processes of GO term enriched in the DEGs that showed reversed regulation between DG and CA regions. **e-f**, Examples of Prdm8 and Hdac5 genes showing differential expression in brain regions visualized by WashU Epigenome Browser.

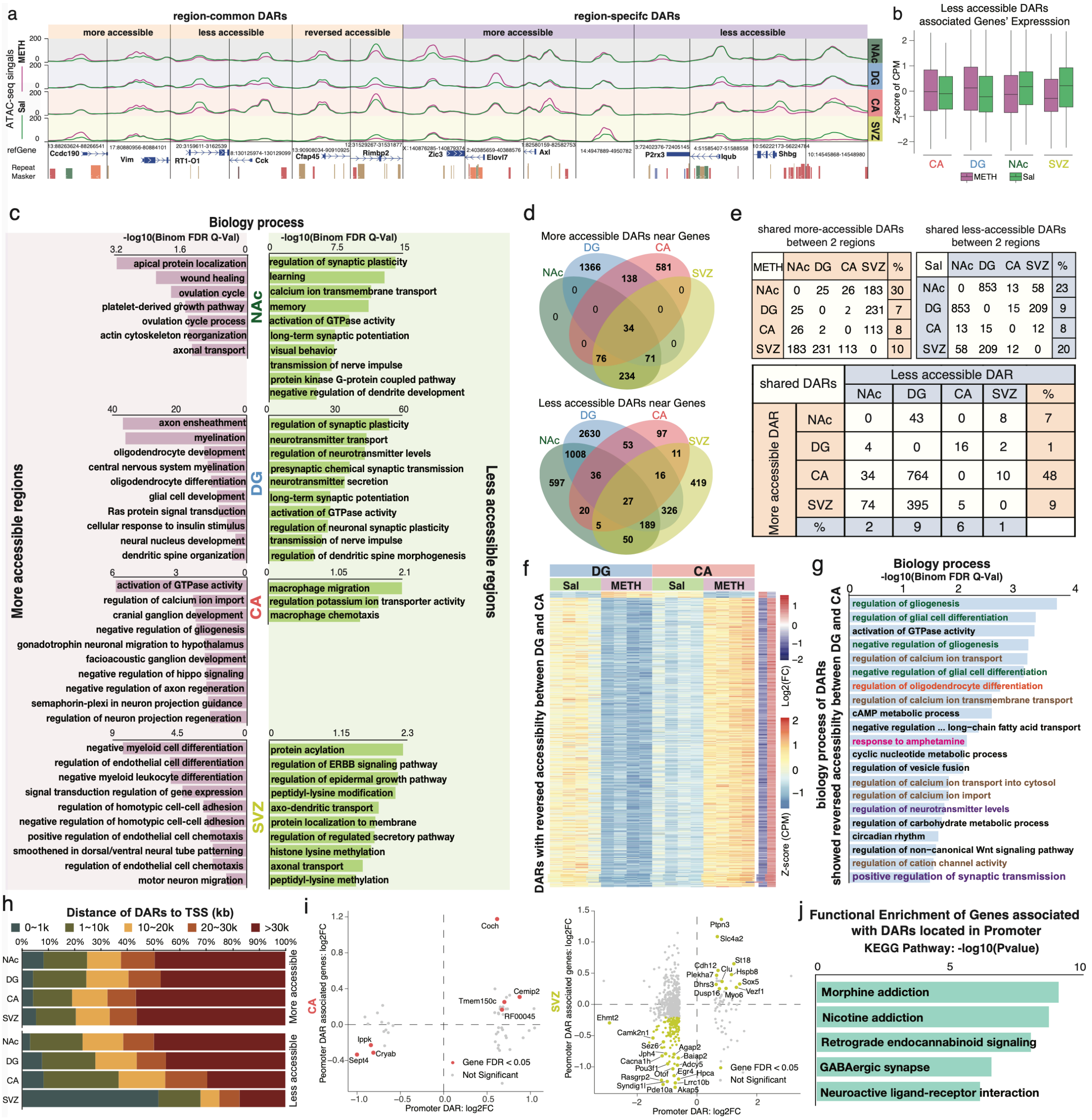

Supplementary Figure 3. The differential accessible regions (DARs) in rat brain regions after binge METH exposure. a, Examples of DARs showed common and specific accessibility in 4 rat brain regions. **b**, Expression (Z-score) of nearest genes assigned to less accessible DARs. The boxplots show the median, upper and lower quartiles, maximums, and minimums. **c**, Enriched biology process of more and less accessible DARs in 4 rat brain regions. **d**, Shared number of genes associated with more and less accessible DARs across 4 rat brain regions. **e**, Number of common DARs between 2 regions separately for more and less accessible DARs, and the number of common DARs between DG and CA showed reversed accessibility. Large percent of more accessible DARs in CA region showed reversed accessibility in DG

region. **f**, Heatmap about Z-score of DARs that displayed reversed accessibility between DG and CA. **g**, Biology processes significantly enriched in DARs showing reversed accessibility between DG and CA. **h**, Distance distribution of DARs to the transcript start sites. **i**, The log2-foldchange distribution of promoter-DARs and those DARs associated genes that assigned the DARs to the nearest genes. **j**, Enriched KEGG pathways based on associated genes of DARs located in the promoter regions.

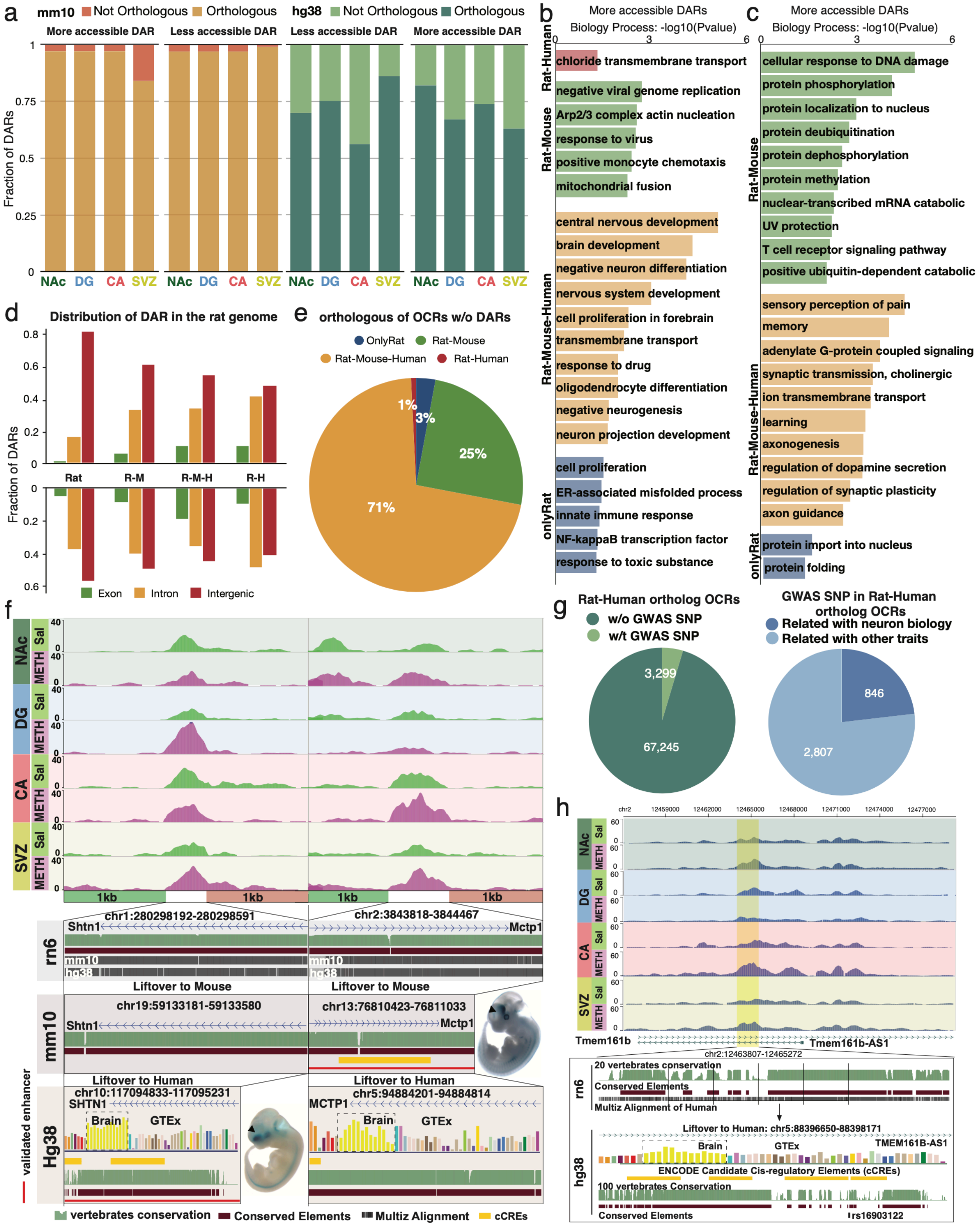

Supplementary Figure 4. Evolutionary conservation of Rat brain DARs responding to binge METH exposure. a, Percent of more and less accessible DARs had orthologous counterparts in mouse and

human genome (mm10 and hg38). **b-c**, Biology processes enriched in genes around (within 20kb) DARs separately for more and less accessible DARs with different conservation status: rat-mouse-human (R-M-H), rat-human (R-H), rat-mouse (R-M) and rat-only (Rat). **d**, Genomic distribution of DARs from 4 different conservation status: exon, intron, and intergenic regions. Large fraction of DARs located in intergenic regions for all 4 groups. **e**, Percent of open chromatin regions (OCRs) without DARs from different conservation status. More than 70% of those OCRs were ortholog in rat, mouse, and human genome. **f**, Two examples of DARs with orthologous interactions with human and mouse validated enhancers. **g**, Number of Rat-Human ortholog OCRs with variants from genome-wide association studies (GWAS SNP) and number of those GWAS SNP associated with neuron biology. **h**, Example of rat-human ortholog DAR overlap with GWAS SNPs associated with neuron biology.

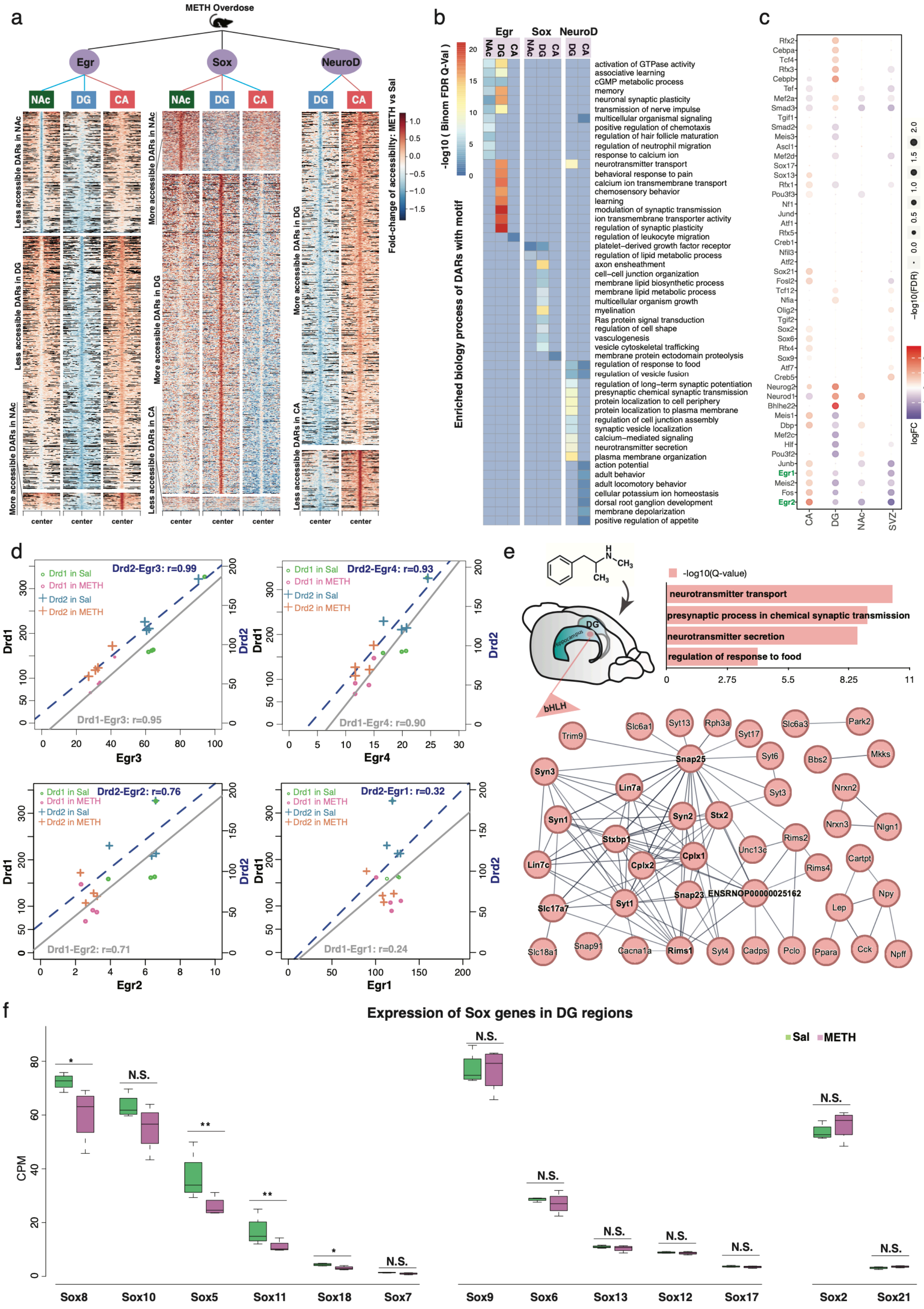

a
METH Overdose
Egr
Sox
NeuroD
NAc
DG
CA
Less accessible DARs in NAc
Less accessible DARs in DG
More accessible DARs in NAc
More accessible DARs in DG
Less accessible DARs in CA
More accessible DARs in DG
Less accessible DARs in DG
More accessible DARs in CA
center
Fold-change of accessibility: METH vs Sal
1.0
0.5
0.0
−0.5
−1.0
−1.5
b
-log10 (Binom FDR Q-Val)
20
15
10
5
0
Enriched biology process of DARs with motif
activation of GTPase activity
associative learning
cGMP metabolic process
memory
neuronal synaptic plasticity
transmission of nerve impulse
multicellular organismal signaling
positive regulation of chemotaxis
regulation of hair follicle maturation
regulation of neutrophil migration
response to calcium ion
neurotransmitter transport
behavioral response to pain
calcium ion transmembrane transport
chemosensory behavior
learning
modulation of synaptic transmission
ion transmembrane transporter activity
regulation of synaptic plasticity
regulation of leukocyte migration
platelet−derived growth factor receptor
regulation of lipid metabolic process
axon ensheathment
cell−cell junction organization
membrane lipid biosynthetic process
membrane lipid metabolic process
multicellular organism growth
myelination
Ras protein signal transduction
regulation of cell shape
vasculogenesis
vesicle cytoskeletal trafficking
membrane protein ectodomain proteolysis
regulation of response to food
regulation of vesicle fusion
regulation of long−term synaptic potentiation
presynaptic chemical synaptic transmission
protein localization to cell periphery
protein localization to plasma membrane
regulation of cell junction assembly
synaptic vesicle localization
calcium−mediated signaling
neurotransmitter secretion
plasma membrane organization
action potential
adult behavior
adult locomotory behavior
cellular potassium ion homeostasis
dorsal root ganglion development
membrane depolarization
positive regulation of appetite
c
Rfx2
Cebpa
Tcf4
Rfx3
Cebpb
Tef
Mef2a
Smad3
Tgif1
Smad2
Meis3
Ascl1
Mef2d
Sox17
Sox13
Rfx1
Pou3f3
Nf1
Jund
Atf1
Rfx5
Creb1
Nfil3
Atf2
Sox21
Fosl2
Tcf12
Nfia
Olig2
Tgif2
Sox2
Sox6
Rfx4
Sox9
Atf7
Creb5
Neurog2
Neurod1
Bhlhe22
Meis1
Dbp
Mef2c
Hlf
Pou3f2
Junb
Egr1
Meis2
Fos
Egr2
CA
DG
NAc
SVZ
−log10(FDR)
0.0
0.5
1.0
1.5
2.0
logFC
−1
0
1
2
d
Drd1 in Sal
Drd1 in METH
Drd2 in Sal
Drd2 in METH
Drd2-Egr3: r=0.99
Drd1-Egr3: r=0.95
Egr3
Drd2-Egr4: r=0.93
Drd1-Egr4: r=0.90
Egr4
Drd2-Egr2: r=0.76
Drd1-Egr2: r=0.71
Egr2
Drd2-Egr1: r=0.32
Drd1-Egr1: r=0.24
Egr1
Drd1
Drd2
e
DG
Hippocampus
bHLH
-log10(Q-value)
neurotransmitter transport
presynaptic process in chemical synaptic transmission
neurotransmitter secretion
regulation of response to food
0
2.75
5.5
8.25
11
Trim9
Slc6a1
Syt13
Rph3a
Syt17
Slc6a3
Park2
Snap25
Syt6
Bbs2
Mkks
Syn3
Lin7a
Syt3
Nrxn2
Syn1
Syn2
Stx2
Nrxn3
Nlgn1
Stxbp1
Rims2
Cplx1
Unc13c
Lin7c
Cplx2
Rims4
Cartpt
Snap23
ENSRNOP00000025162
Npy
Slc17a7
Syt1
Lep
Slc18a1
Snap91
Cacna1a
Rims1
Syt4
Cadps
Pclo
Ppara
Cck
Npff
f
Expression of Sox genes in DG regions
Sal
METH
CPM
0
20
40
60
80
*
N.S.
**
Sox8
Sox10
Sox5
Sox11
Sox18
Sox7
Sox9
Sox6
Sox13
Sox12
Sox17
Sox2
Sox21

Supplementary Figure 5. METH exposure **induced DARs-associated regulatory networks in rat brain.** **a**, Heatmap of more and less accessible DARs contained the Egr, Sox and NeuroD binding motifs in different brain regions. b, Enriched biology processes based on DARs with Egr, Sox and NeuroD binding motifs. **c**, The binge METH induced expression changes of transcription factors (TFs) whose binding motifs enriched in DARs. **d**, The correlation of expression changes between Drd1/2 genes and Egr family genes in rat NAc region with Meth stimulus. **e**, Gene regulatory network and enriched biology process built by genes around DARs with bHLH binding motif in DG. **f**, Expression changes of Sox genes in DG region responding to binge METH exposure. The boxplots show the median, upper and lower quartiles, maximums, and minimums.

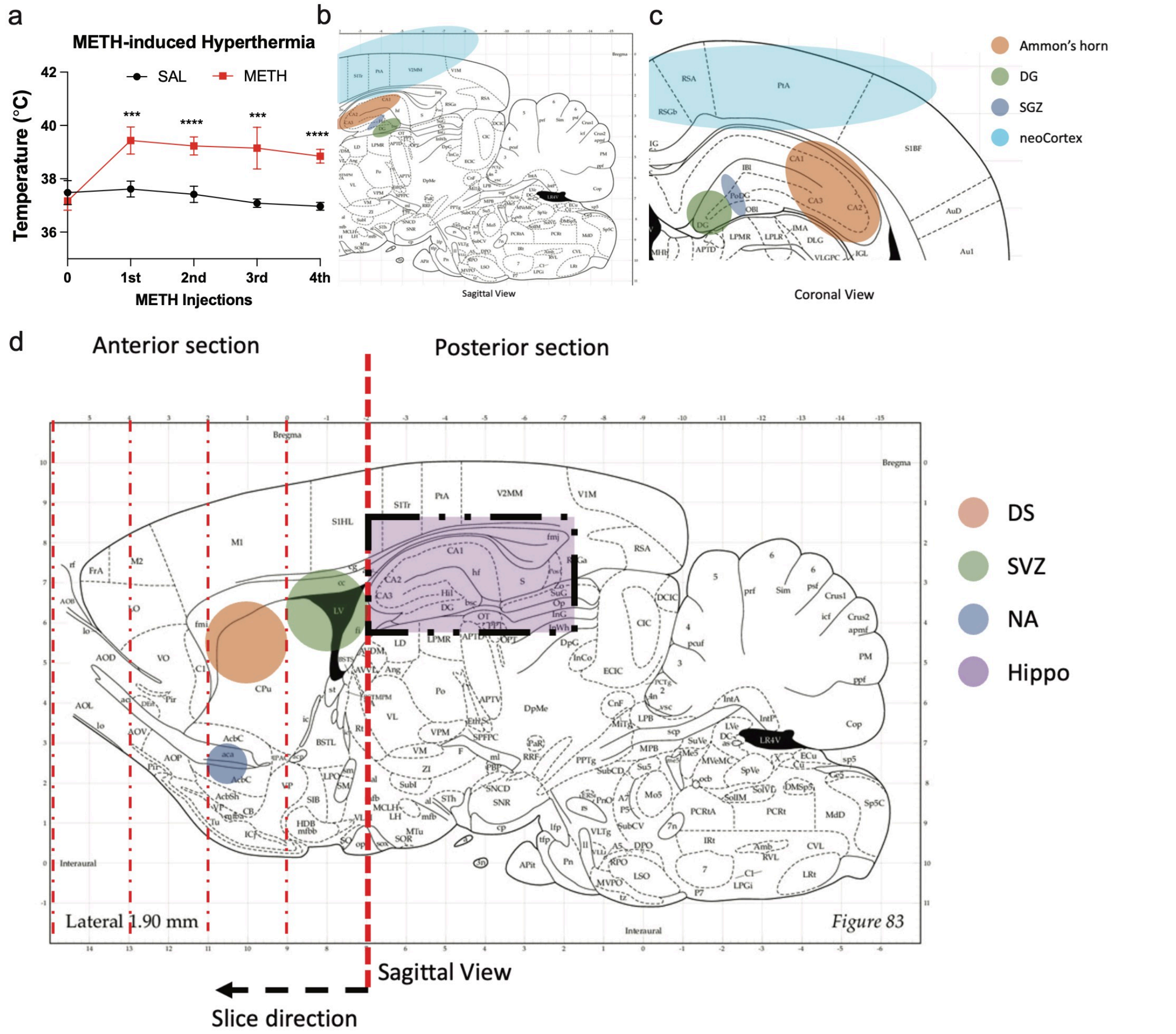

Supplementary Figure 6. Experiment of 4 Rat brain regions. a, The temperature (°C) of Rat after the METH injections. The error bars are standard errors of the mean. **b-d**, different sections of rat brain regions. The original section plots of brain regions from online tool developed by Gaidica Lab (http://labs.gaidi.ca/rat-brain-atlas/).
