# Supplementary material for: Methamphetamine-induced region-specific transcriptomic and epigenetic changes in the brain of male rats
Source: Commun Biol. 2023 Sep 27;6:991. doi: 10.1038/s42003-023-05355-3 (PMC10533900; doi:10.1038/s42003-023-05355-3)
Supplement: Supplementary file 2 — Description of Additional Supplementary Files [file 42003_2023_5355_MOESM2_ESM.pdf]

Description of Additional Supplementary Files

File name: Supplementary Data 1
Description: METH binge and saline samples of 4 rat brain regions for RNA-seq and ATAC-seq data with quality control parameters and RIN information**.**

File name: Supplementary Data 2
Description: Region-specific genes and open chromatin regions (OCRs) identified in 4 normal brain regions.

File name: Supplementary Data 3
Description: GO terms enriched in region-specific genes and open chromatin regions of 4 normal rat brain regions.

File name: Supplementary Data 4
Description: Differential expressed genes (DEGs) identified in 4 rat brain regions responding to binge METH exposure.

File name: Supplementary Data 5
Description: Enriched biology processes separately based on up and down-regulated DEGs in 4 rat brain regions.

File name: Supplementary Data 6
Description: Binge METH exposure induced differential accessible regions (DARs) in 4 rat brain regions.

File name: Supplementary Data 7
Description: Biology processed enriched in more and less accessible DARs of 4 rat brain regions.

File name: Supplementary Data 8
Description: Distribution of DARs in 4 rat brain regions. Number of more and less accessible DARs located in genomic promoter, exon, intron and intergenic regions. Number of mouse orthologous regions of rat brain DARs with cis-regulatory elements including CTCF-only, DNase-H3K4me3, promoter, proximal enhancer and distal enhancer.

File name: Supplementary Data 9
Description: Enriched mouse phenotypes of orthologous DARs with cis-regulatory annotations.

File name: Supplementary Data 10
Description: Number of orthologous DARs in rat, mouse and human genomes.

File name: Supplementary Data 11
Description: More and less accessible DARs had ortholog interactions with validated enhancers of human and mouse from VISTA Enhancer Browser.

File name: Supplementary Data 12
Description: The human-rat ortholog regions of rat brain OCRs contained the GWAS SNPs associated with neuron biology.

File name: Supplementary Data 13
Description: Transcription factors binding motifs enriched in more and less accessible DARs of 4 brain regions.

File name: Supplementary Data 14
Description: DARs contained the transcription factors binding motifs in 4 rat brain regions.

File name: Supplementary Data 15
Description: GO enrichment analysis of DARs contained different transcription factors binding motifs.
