# Supplementary material for: Methamphetamine-induced region-specific transcriptomic and epigenetic changes in the brain of male rats
Source: Commun Biol. 2023 Sep 27;6:991. doi: 10.1038/s42003-023-05355-3 (PMC10533900; doi:10.1038/s42003-023-05355-3)
Supplement: Supplementary file 18 — Reporting Summary [file 42003_2023_5355_MOESM18_ESM.pdf]

nature portfolio

Corresponding author(s): Anna Moszczynska
Bo Zhang

Last updated by author(s): Jul 14, 2023

Reporting Summary

Nature Portfolio wishes to improve the reproducibility of the work that we publish. This form provides structure for consistency and transparency in reporting. For further information on Nature Portfolio policies, see our Editorial Policies and the Editorial Policy Checklist.

Statistics

For all statistical analyses, confirm that the following items are present in the figure legend, table legend, main text, or Methods section.

n/a	Confirmed	
☐	☒	The exact sample size (*n*) for each experimental group/condition, given as a discrete number and unit of measurement
☐	☒	A statement on whether measurements were taken from distinct samples or whether the same sample was measured repeatedly
☐	☒	The statistical test(s) used AND whether they are one- or two-sided<br>*Only common tests should be described solely by name; describe more complex techniques in the Methods section.*
☐	☒	A description of all covariates tested
☐	☒	A description of any assumptions or corrections, such as tests of normality and adjustment for multiple comparisons
☐	☒	A full description of the statistical parameters including central tendency (e.g. means) or other basic estimates (e.g. regression coefficient) AND variation (e.g. standard deviation) or associated estimates of uncertainty (e.g. confidence intervals)
☐	☒	For null hypothesis testing, the test statistic (e.g. *F*, *t*, *r*) with confidence intervals, effect sizes, degrees of freedom and *P* value noted<br>*Give P values as exact values whenever suitable.*
☒	☐	For Bayesian analysis, information on the choice of priors and Markov chain Monte Carlo settings
☒	☐	For hierarchical and complex designs, identification of the appropriate level for tests and full reporting of outcomes
☐	☒	Estimates of effect sizes (e.g. Cohen's *d*, Pearson's *r*), indicating how they were calculated

Our web collection on statistics for biologists contains articles on many of the points above.

Software and code

Policy information about availability of computer code

Data collection	No software was used for data collection
Data analysis	ATAC-seq data were processed by AIAP package<br>RNA-seq data were processed by Cutadapt (v2.7; --quality-cutoff=15,10 --minimum-length=36), FastQC (v0.11.4), and STAR (v2.5.2b; --quantMode TranscriptomeSAM --outWigType bedGraph --outWigNorm RPM) to do the trimming, QC report and rat genome mapping (rn6)<br>EdgeR was used for differential analysis<br>GREAT (version 4.0.0) and DAVID (v6.8) used for GO analysis<br>WashU Epigenome Browser (http://epigenomegateway.wustl.edu/) used for data visualization<br>HOMER software (v4.11.1) used for transcription factors binding motifs enrichment analysis

For manuscripts utilizing custom algorithms or software that are central to the research but not yet described in published literature, software must be made available to editors and reviewers. We strongly encourage code deposition in a community repository (e.g. GitHub). See the Nature Portfolio guidelines for submitting code & software for further information.

Data

Policy information about availability of data

All manuscripts must include a data availability statement. This statement should provide the following information, where applicable:

- Accession codes, unique identifiers, or web links for publicly available datasets
- A description of any restrictions on data availability
- For clinical datasets or third party data, please ensure that the statement adheres to our policy

All sequencing data can be downloaded from Gene Expression Omnibus (GEO): GSE199374

Research involving human participants, their data, or biological material

Policy information about studies with human participants or human data. See also policy information about sex, gender (identity/presentation), and sexual orientation and race, ethnicity and racism.

Reporting on sex and gender	N/A
Reporting on race, ethnicity, or other socially relevant groupings	N/A
Population characteristics	N/A
Recruitment	N/A
Ethics oversight	N/A

Note that full information on the approval of the study protocol must also be provided in the manuscript.

Field-specific reporting

Please select the one below that is the best fit for your research. If you are not sure, read the appropriate sections before making your selection.

☒ Life sciences ☐ Behavioural & social sciences ☐ Ecological, evolutionary & environmental sciences

For a reference copy of the document with all sections, see nature.com/documents/nr-reporting-summary-flat.pdf

Life sciences study design

All studies must disclose on these points even when the disclosure is negative.

Sample size	Total 32 RNA-seq samples from 4 rat brain regions, Nucleus accumbens (NAc), Dentate gyrus (DG), Ammon's horn (CA) and Subventricular zone (SVZ). And each region had 4 saline sample as control and 4 METH exposures samples<br>Total 29 ATAC-seq samples from 4 rat brain regions:<br>1) NAc: 4 Saline X 4 METH exposures samples<br>2) DG: 4 Saline X 4 METH exposures samples<br>3) CA: 4 Saline X 4 METH exposures samples<br>4) SVZ: 3 Saline X 2 METH exposures samples
Data exclusions	No data was excluded
Replication	see Sample size for replication information
Randomization	No randomization
Blinding	No blinding

Reporting for specific materials, systems and methods

We require information from authors about some types of materials, experimental systems and methods used in many studies. Here, indicate whether each material, system or method listed is relevant to your study. If you are not sure if a list item applies to your research, read the appropriate section before selecting a response.

Materials & experimental systems

n/a	Involved in the study
☒	☐ Antibodies
☒	☐ Eukaryotic cell lines
☒	☐ Palaeontology and archaeology
☐	☒ Animals and other organisms
☒	☐ Clinical data
☒	☐ Dual use research of concern
☒	☐ Plants

Methods

n/a	Involved in the study
☒	☐ ChIP-seq
☒	☐ Flow cytometry
☒	☐ MRI-based neuroimaging

Animals and other research organisms

Policy information about studies involving animals; ARRIVE guidelines recommended for reporting animal research, and Sex and Gender in Research

Laboratory animals	Adult male Sprague-Dawley rats (Harlan, Indianapolis, IN, USA) (weighing 250–300 g on arrival)
Wild animals	N/A
Reporting on sex	Male
Field-collected samples	N/A
Ethics oversight	All animal procedures were conducted in strict accordance with the National Institutes of Health (NIH) Guide for the Care and Use of Laboratory Animals and were approved by the Institutional Animal Care and Use Committee (IACUC) at Wayne State University. The description of animal procedures meets the ARRIVE recommended guidelines described by The National Centre for the Replacement, Refinement and Reduction of Animals in Research

Note that full information on the approval of the study protocol must also be provided in the manuscript.
